# Genome-wide chromatin accessibility landscape and dynamics of transcription factor networks during ovule and fiber development in cotton

**DOI:** 10.1186/s12915-023-01665-4

**Published:** 2023-07-31

**Authors:** Yu Bao, Yangyang Wei, Yuling Liu, Jingjing Gao, Shuang Cheng, Guanqing Liu, Qi You, Peng Liu, Quanwei Lu, Pengtao Li, Shulin Zhang, Nan Hu, Yangshuo Han, Shuo Liu, Yuechao Wu, Qingqing Yang, Zhaoguo Li, Guowei Ao, Fang Liu, Kunbo Wang, Jiming Jiang, Tao Zhang, Wenli Zhang, Renhai Peng

**Affiliations:** 1 Jiangsu Key Laboratory of Crop Genomics and Molecular Breeding/Jiangsu Key Laboratory of Crop Genetics and Physiology/Jiangsu Co-Innovation Center for Modern Production Technology of Grain Crops, Agricultural College of Yangzhou University, Yangzhou, 225009 China; 2 Anyang Institute of Technology, Anyang, Henan 455000 China; 3 Key Laboratory of Plant Functional Genomics of the Ministry of Education/Joint International Research Laboratory of Agriculture and Agri-Product Safety of Ministry of Education of China, Yangzhou University, Yangzhou, 225009 China; 4 Research Base, Anyang Institute of Technology, State Key Laboratory of Cotton Biology, Anyang, Henan 455000 China; 5 National Key Laboratory for Crop Genetics and Germplasm Enhancement and Utilization, Collaborative Innovation Center for Modern Crop Production Co-Sponsored By Province and Ministry (CIC-MCP), Nanjing Agricultural University, No.1 Weigang, Nanjing, 210095 Jiangsu China; 6 Zhengzhou University, Zhengzhou, Henan 450001 China; 7 Institutes of Agricultural Science and Technology Development, Joint International Research Laboratory of Agriculture and Agri-Product Safety, Yangzhou University, Yangzhou, 225009 China; 8 Department of Plant Biology, Michigan State University, East Lansing, MI USA; 9 Department of Horticulture, Michigan State University, East Lansing, MI USA; 10 Michigan State University AgBioResearch, East Lansing, MI USA

**Keywords:** Cotton fiber, DNase-seq, Network, *Cis*-regulatory DNA elements, Transcription factors

## Abstract

**Background:**

The development of cotton fiber is regulated by the orchestrated binding of regulatory proteins to *cis*-regulatory elements associated with developmental genes. The *cis–trans* regulatory dynamics occurred throughout the course of cotton fiber development are elusive. Here we generated genome-wide high-resolution DNase I hypersensitive sites (DHSs) maps to understand the regulatory mechanisms of cotton ovule and fiber development.

**Results:**

We generated DNase I hypersensitive site (DHS) profiles from cotton ovules at 0 and 3 days post anthesis (DPA) and fibers at 8, 12, 15, and 18 DPA. We obtained a total of 1185 million reads and identified a total of 199,351 DHSs through ~ 30% unique mapping reads. It should be noted that more than half of DNase-seq reads mapped multiple genome locations and were not analyzed in order to achieve a high specificity of peak profile and to avoid bias from repetitive genomic regions. Distinct chromatin accessibilities were observed in the ovules (0 and 3 DPA) compared to the fiber elongation stages (8, 12, 15, and 18 DPA). Besides, the chromatin accessibility during ovules was particularly elevated in genomic regions enriched with transposable elements (TEs) and genes in TE-enriched regions were involved in ovule cell division. We analyzed *cis*-regulatory modules and revealed the influence of hormones on fiber development from the regulatory divergence of transcription factor (TF) motifs. Finally, we constructed a reliable regulatory network of TFs related to ovule and fiber development based on chromatin accessibility and gene co-expression network. From this network, we discovered a novel TF, WRKY46, which may shape fiber development by regulating the lignin content.

**Conclusions:**

Our results not only reveal the contribution of TEs in fiber development, but also predict and validate the TFs related to fiber development, which will benefit the research of cotton fiber molecular breeding.

**Supplementary Information:**

The online version contains supplementary material available at 10.1186/s12915-023-01665-4.

## Background

In eukaryotes, gene expression is regulated by the interaction between *trans*-acting factors and *cis*-regulatory DNA elements (CREs). CREs are short, non-coding sequences that contain binding motifs of transcription factors (TFs) and other *trans*-acting factors. Most common classes of CREs include promoters, enhancers, insulators, and silencers. Thus, knowledge of which *trans*-acting factors, as well as when and where these *trans*-acting factors bind to CREs, is critical for understanding the regulation and function of a given gene. Accessible chromatin regions (ACRs) are nucleosome-depleted chromosomal regions that harbor CREs [1]. DNase I hypersensitive sites (DHSs) are genomic regions that exhibit hypersensitivity to DNase I cleavage [2]. DNase-seq, a high-throughput method that identifies DHSs, has been used as an effective tool to identify genome-wide CREs in animals and plants [3–13]. In *Arabidopsis*, DHSs were successfully used to predict the genomic locations of transcriptional enhancers [11, 14]. In tomato, in a comparison of DNase-seq data during tomato development and the ripening stage, it was found that several major genes involved in ascorbic acid biosynthesis may be coordinated and regulated by the same transcription factor [7]. In maize, DNase-seq was used to reveal how transposable element (TE)-derived DHSs (teDHSs) influence gene expression [15, 16]. In addition, DNase-seq has been widely used to study the characteristics of TF binding among different species, to build regulatory networks, and to study the relationship between *cis*-regulatory divergence and domestication in the evolutionary process [6, 8–10, 17, 18].

The most widely cultivated cotton species, *Gossypium hirsutum* L. (AADD, 2*n* = 4*x* = 52), is an allotetraploid and provides the largest source of renewable textile fiber. Cotton also serves as a model species for polyploidy studies [19, 20]. A cotton fiber is a single cell derived from the ovule epidermis and undergoes a sequence of four successive developmental stages over its entire lifespan: fiber initiation (− 3 to 3 DPA), cell elongation (3 to 23 DPA), secondary wall deposition (SCW) (20 to 40 DPA), and maturation (40 to 50 DPA) [21]. On the day post anthesis, fiber initiation was observed on the epidermal surface of the ovule, followed by cell elongation. The elongation of fibers is rapid, independent of cell division, and appears in the period of 3–20 DPA. As the fiber elongates and diameter increases, so too does the cell wall become thicker. Secondary cell wall deposition occurs from 15 to 40 DPA, followed by fiber maturation from 40 to 50 DPA. Fiber cell initiation and elongation determine cotton fiber number and fiber length, which are the major factors determining lint yield. Over the past decades, a great number of researches have focused on understanding the mechanisms that regulate fiber development. Epigenetic modifications are important regulators in plant development and research on epigenetics of cotton fiber development become more and more extensive. By comparing small RNA data between ovules (− 3, 0, 3 DPA) and fibers (7, 12, 20, 25 DPA), Liu et al. unraveled miRNA156/157 plays an essential role in fiber elongation [22]. Small RNA deep sequencing from ovules (− 1, 0, 1, 3, 5 DPA) analysis revealed that the bidirectional transcript of *GhMML3_A12* generated the siRNA involved in cotton fiber initiation [23]. Using the fibreless mutant xuzhou142fl, the lncRNAs and circular RNAs were identified from epidermal cells of ovules with attached fibers at 0 and 5 DPA, and three lncRNAs were shown to function during fiber development [24]. Song et al. uncovered the fiber cells (15 DPA) generated additional heterochromatic CHH hypermethylation independent of RdDM compared with ovules (0 DPA), which repressed TEs and nearby genes [25]. Through comparison with fiber initiation (ovules of 0 DPA), the increase of DNA methylation in fiber elongation (fibers of 10, 20, and 30 DPA) was mainly dependent on an active H3K9me2-dependent pathway and the dynamic DNA methylation affects lipid biosynthesis and reactive oxygen species levels during fiber differentiation [26]. Recently, Pei et al. described the dynamic changes of the 3D structures of the subgenome and uncovered their regulatory implications on gene expression in different stages of fiber development (ovules of 0 DPA; fibers of 5, 10, and 20 DPA) [27]. Although the studies of cotton fiber cell differentiation have made remarkable progress in the epigenetically mediated transcriptional program, little is known about the overall *cis* and *trans* regulatory landscape during fiber development.

In this study, we generated genome-wide high-resolution DHSs maps related to cotton ovules and fiber elongation. By integrating DNase-seq with ChIP-seq and RNA-seq data generated from cotton ovules and fibers, we unveiled dynamic changes in gene regulation between ovules and fibers. We determined activating and repressing *cis*-regulatory elements by calculating the correlation between RNA-seq and DNase-seq data and revealed the regulatory differential motifs and their downstream targets during different developmental periods. This study provides a valuable resource for understanding the regulatory mechanisms of cotton ovule and fiber development, thereby facilitating the engineering of high-quality cotton fiber via genetic modification of the corresponding regulatory elements.

## Results

### Profiling of DHSs associated with fiber development in G. hirsutum

To investigate the regulatory DNA landscape during cotton fiber development, we developed DNase-seq libraries of cotton ovules at 0 and 3 DPA (days post anthesis) and fiber cells during elongation at 8, 12, 15, and 18 DPA [28] (Additional file 1: Fig. S1A). Pearson correlation coefficients between the biological replicates ranged from 0.95 to 0.99 and the rescue ratio ranging from 0.71 to 0.95, indicating that the data sets are highly reproducible and that the identified DHSs are indeed reliable [29] (Additional file 2: Table S1). Across the cotton genome, we identified a total of 33,576 DHSs from 0 DPA, 57,501 from 3 DPA, 94,636 from 8 DPA, 93,072 from 12 DPA, 117,136 from 15 DPA, and 102,326 from 18 DPA tissues (Additional file 2: Table S1). Recently, Pei et al. have shown that the amount of histone modifications during the ovule stage is significantly less than that during the fiber elongation, such as activating modifications H3K27ac and H3K4me3 [27]. Similarly, the number of DHSs in the ovules was much smaller than that in the fibers, suggesting a significant difference in epigenetic modifications between the ovule and fiber periods.

**Fig. 1:**
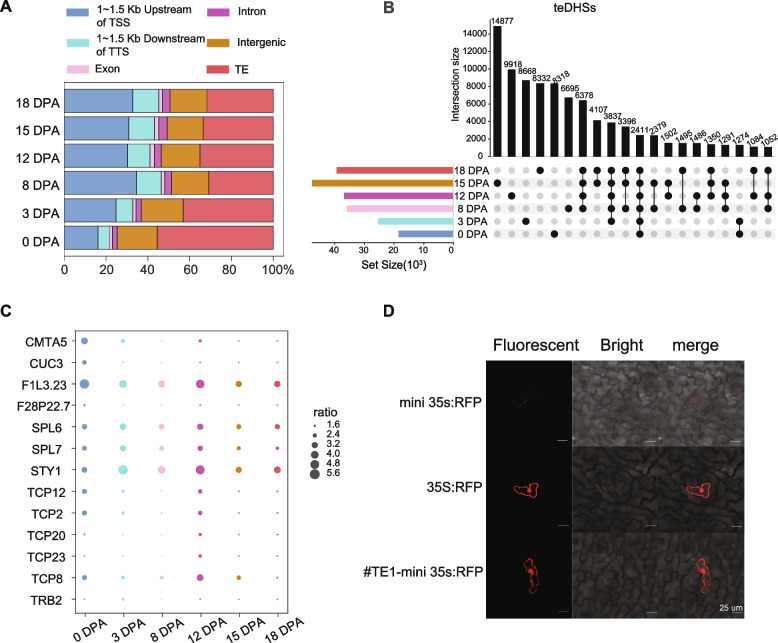
Distribution of DHSs in the cotton genome and identification of teDHSs. **A** Distribution of DHSs in the cotton genome. The *x-*axis shows the percentage of DHSs associated with each type of genomic location. **B** UpSet plot of teDHSs during fiber development. The left bars show the number of total teDHSs at different periods. The right bar plot and points represent the number of teDHSs in specific intersection. **C** Motifs were significantly enriched in teDHS. We calculated the ratio of means of significant enrichment motifs during fiber development in teDHSs to those in non-teDHSs. **D** Fluorescent, bright-field and merge (bright-field and red fluorescent) confocal laser scanning micrographs of cotton leaf cells bombarded with #TE1-mini35s:RFP construct DNA-loaded gold particles are presented. 35S:RFP is a positive control with a construct with RFP expression driven by the 35 s promoter. mini 35 s:RFP is a negative control with a construct with RFP expression driven by the mini 35 s promoter. #TE1-mini35s:RFP is a construct of validation of enhancer function, which conducted using a construct with RFP expression driven by the mini 35 s promoter and teDHSs. RFP expression in the constructs with teDHSs indicates that the teDHSs promote gene expression. Scale bar, 25 µm

We further investigated the differences in the genomic distribution of ovule and fiber DHSs. At the elongation stage, over 30.4% of the DHSs were located within 1.5 kb upstream of transcription start sites (TSSs), the putative promoter regions (Fig. 1A). However, only 16.2% of the DHSs were located in the promoter regions at 0 DPA (Fig. 1A), which was significantly lower than at elongation stage (*p*-value < 2.2 × 10^−16^, Fisher’s exact test). Similarly, the proportion of DHSs located in the 1.5 kb downstream of transcription termination sites (TTSs) in the ovules was significantly lower than that during the fiber elongation period. Interestingly, highly variable percentages of DHSs were located in TE regions during different development stages, ranging from 30.9% at 8 DPA to 55.4% at 0 DPA. Taken together, DHSs were found to be enriched in gene promoters and TE regions. From ovule to fiber, the proportion of DHSs in the TE regions was significantly reduced and the proportion of DHSs in the promoter regions was significantly increased, this suggested that gene expression and regulation landscapes are strikingly different between ovules and fiber elongation.

### TEs play important roles in the distribution of DHSs across cotton genomes

Epigenomic studies of different cell types and organisms have shown that TEs are an important source of regulatory elements. TEs contribute nearly half of the active elements in the human genome, and as much as 63% of open chromatin regions are associated with TEs in primates [30] and more than 50% in maize [16]. Recently, Han et al. revealed that TEs promotes the formation of DHS in polyploidization in cotton. In leaves, 56% of *Gossypium hirsutum*-specific DHSs are derived from TE-related sequence [31]. By scanning the cotton genome for TE-derived DHSs (teDHSs), we identified 18,424, 25,331, 35,820, 36,826, 47,582, and 39,331 teDHSs at 0, 3, 8, 12, 15, and 18 DPA, respectively (Fig. 1B). Of these, 54.9% DHSs were identified as teDHSs at 0 DPA and approximately 40% DHSs were identified as teDHSs during fiber elongation. In addition, only 6% of the teDHSs were located in the promoter regions of the gene (within 1.5 kb upstream of a TSS) at 0 DPA, while the proportion increased to 28% at 8 DPA. In general, the teDHSs were mostly located outside of promoter regions. The distance between a teDHS and its closest gene in ovules (mean distance of 33.7 kb) was significantly greater than that at the fiber elongation stage (mean distance of 11.1 kb) (*p*-value < 2.2 × 10^−16^, K-S test) (Additional file 1: Fig. S1B).

In maize, long terminal repeat (LTR) retrotransposons harbor the largest number of “TE-derived enhancers,” followed by helitrons and terminal inverted repeat (TIR) transposons [16]. Previous studies have shown that TEs play an important role in the distribution of ACR, especially class II TE [32]. We found that *Gypsy* retrotransposons were associated with the largest number of teDHSs (*Gypsy*-teDHSs) at all six stages, followed by *Copia* retrotransposons. Interestingly, *Gypsy*-teDHSs accounted for as much as 58.9% of teDHSs at 0 DPA, while the proportion decreased to 25.7% at 8 DPA; similarly, the *MuDR* family was significantly reduced from 2.9 to 1.9%. We used hypergeometric distribution to identify the enrichment of TE subclasses at each stage. The results showed that the teDHSs were significantly enriched within *LINE* at 3 DPA and the fiber elongation and were strongly enriched within *Gypsy* and *MuDR* at the ovules and 12 DPA (Additional file 1: Fig. S1C).

To examine the regulatory pathways that the teDHSs may participate in, we analyzed the enrichment of TF-binding motifs in the teDHSs. A series of enriched motifs were found to be relevant to ovules and fiber elongation stages (Fig. 1C). For example, motifs enriched in the teDHSs in ovules were found related to TFs involved in stress response (*CMTA5*) [33] and ovule development (*CUC3*) [34]. Motifs enriched in the teDHSs at all stages were found to be related to TFs involved in induction of auxin biosynthesis (*STY1*) [35], participation in ovule development (*TCP2*) [36], and regulation of cell cycle control and ribosomal protein genes (*TCP20*) [37, 38] (Fig. 1C). These results support that these teDHSs are bona fide functional CREs, such as enhancers, in regulating gene expression during ovules and fiber elongation. As expected, the expression level of genes with teDHSs is significantly higher than those without DHSs (*p*-value < 2.2 × 10^−16^, K-S test) (Additional file 1: Fig. S1D). To further explore this hypothesis, 12 teDHSs were selected for functional assays (Additional file 3: Table S2). We employed a fluorescent reporter gene assay in cotton leaves to validate the enhancer activity of these teDHSs. The target teDHS sequences were inserted into the pCAMBIA2300 vector that contains a minimal CaMV 35S promoter and an RFP (red fluorescent protein) reporter gene. Constructs with the mini35s promoter and the 35S promoter were used as the negative and positive controls, respectively. Reporter gene expression was detected from 8 of the 12 teDHSs (66.7%) in the assays (Fig. 1D; Additional file 1: Fig. S2).

### Dynamics of chromatin accessibility between ovules and fiber elongation

**Fig. 2:**
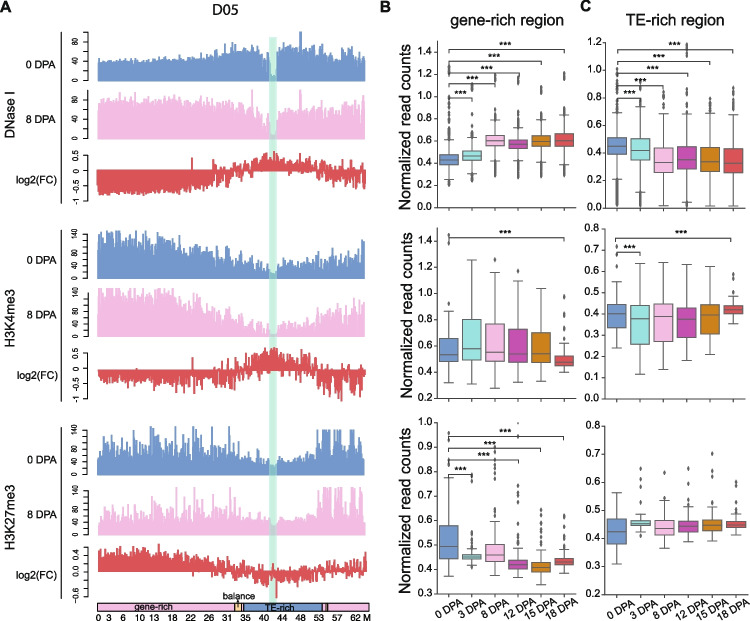
Genome-wide mapping of chromatin accessibility and histone modification in cotton. **A** Distribution of DNase-seq, H3K4me3, and H3K27me3 along chromosome D05 in 0 and 8 DPA. The *y*-axis shows normalized read counts in 100-kb windows. FC: fold change of 0 DPA reads count with respect to 8 DPA reads count. The green shade marks the location of the centromeric on chromosome D05. The bottom bar shows the division of the D05 chromosome: pink: gene-rich region; dark blue: TE-rich region; yellow: balance region. **B**,** C** Chromatin accessibility, H3K4me3 and H3K27me3 modification in gene-enriched regions (**B**) and TE-enriched regions (**C**) across different fiber development stages. Statistical significances were calculated by Kolmogorov–Smirnov (K-S) test (***p < 0.001)

There are large-scale changes in open chromatin dynamics between ovules and fibers (Fig. 1A). To better evaluate the open chromatin dynamics during the two development stages, we visualized the genome-wide distribution of DNase-seq profiles as well as the distribution of genes and TEs. We divided the chromosomes into three groups (gene-enriched regions, TE-enriched regions and balance regions) based on the proportion of genes/TEs in 1 Mb windows (Additional file 1: Fig. S3B). As we expected, different distribution patterns of chromatin accessibility were observed between the two development stages, such as chromosome D05 (Fig. 2A; Additional file 1: Fig. S3C). At 8 DPA, the lowest DHS density was observed around the centromere of chromosome D05. The DHS density tended to increase toward the distal region of the chromosome. At 0 DPA, in contrast, the distribution of DHSs exhibited a different trend: the highest DHS densities were observed in the pericentromeric regions of chromosome D05 (Fig. 2A; Additional file 1: Fig. S3C and S4). We further compared the changes in chromatin accessibility in gene-enriched regions at different stages. Chromatin accessibility in gene-enriched regions during fiber elongation was significantly higher than that in ovules (Fig. 2B). In contrast to the results in gene-enriched regions, we found that the cotton ovules showed higher chromatin accessibility than the fiber elongation stages in TE-enriched regions (Fig. 2C). Our results agree with a previous study that reported higher chromatin accessibility at 0 DPA than 10 DPA, 20 DPA, and 30 DPA in long TEs (> 4 kb) and pericentromeric regions [26]. The centromere region is embedded in the TE-enriched region. We further examined dynamics of gene expression and open chromatin modification in centromere regions during fibro development. However, the results showed that most of the genes were not expressed in the ovule and fiber stage, and there was no epigenetic modification (Additional file 4: Table S3).

We generated ChIP-seq data for H3K4me3 and H3K27me3, which are positively and negatively correlated with gene expression, respectively (Additional file 1: Fig. S3A). Unlike the DNase-seq results, both H3K4me3 and H3K27me3 are enriched at the distal parts of the chromosome and depleted around the centromere (Fig. 2A; Additional file 1: Fig. S3D and S3E). By comparing the dynamics of histone modifications on chromosomes during ovules and fiber elongation, the changes of H3K4me3 were found to be consistent with the changes in Dnase-seq (Fig. 2A; Additional file 1: Fig. S4). As expected, the dynamics of H3K27me3 modification showed the opposite trend (Fig. 2A; Additional file 1: Fig. S4). Although we can detect changes in histone modifications during ovules and fiber elongation, H3K4me3 showed no significant variations in the gene-rich regions and TE-rich regions; H3K27me3 gradually decreased in the gene-rich regions, but did not change significantly in the TE-rich regions (Fig. 2B, C). These results implied that dynamic changes in epigenetic marks, especially chromatin accessibility and H3K27me3, play important roles in cotton fiber development.

### Involvement of chromatin accessibility in pericentromeric regions in the ovule and fiber stages

**Fig. 3:**
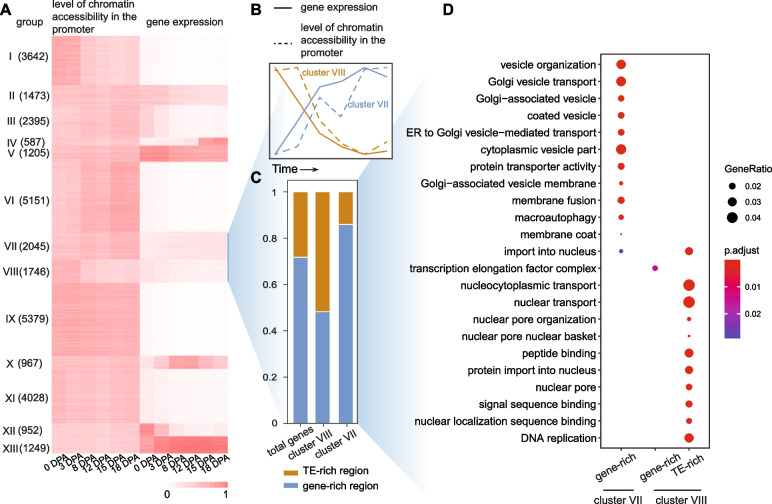
Involvement of chromatin accessibility in pericentromeric regions in the ovule and fiber stages. **A** Grouping of genes by the pattern of DHSs in promoter and closest gene expression. All expressed gene were categorized into 13 clusters (I to XIII). The numbers in brackets represent the number of genes in each cluster. Gene expression values and level of chromatin accessibility in the promoter, normalized to 0–1 with the 95th quantile value of each column, are indicated with a white to red graded color. **B** Relative transcript level and promoter DHSs changes for cluster VII and VIII genes. **C** Distribution pattern of all genes, cluster VII and cluster VIII genes on the genome. Cluster VII gene is significantly enriched in TE-rich region and cluster VIII gene is significantly enriched in gene-rich region. **D** Gene Ontology (GO) terms with significant enrichment levels were present for clusters VII and VIII

Gene expression undergoes dynamic changes during cotton fiber development. The dynamic DNase-seq maps of cotton ovules and fibers provided insights into understanding the regulatory relationship between gene expression and chromatin accessibility. We measured the expression levels of genes in six stages and examined the level of chromatin accessibility in the promoter of the genes. A *k*-means clustering analysis categorized genes into 13 clusters (Fig. 3A). Among the 13 clusters, 3791 genes classified in cluster VII and cluster VIII showed coordinated changes between gene expression and chromatin accessibility. Although cluster I, III, VI, and IX display similar or opposite changing trends, these clusters have been overlooked due to their minor magnitude changes (rangeability < 0.05) in gene expression or open chromatin (Additional file 1: Fig. S5A). Clusters III and VI exhibit low levels of open chromatin during the ovule stage, but they are enriched with the active H3K4me3 modification. This could be one of the reasons for the higher expression levels of clusters III and VI during the ovule stage (Additional file 1: Fig. S5B). In cluster VIII, genes have a higher expression level in the ovules than that in the fiber elongation stages. Higher level of chromatin accessibility in the promoter of cluster VIII genes in the ovules and the level gradually decreased during fiber development (Fig. 3B). These results indicated that cluster VIII genes may be specific for ovules and are closely related to chromatin accessibility changes between ovules and fibers. Interestingly, we found that cluster VIII genes account for 51.8% of the TE-enriched regions, which is significantly higher than the frequency of genes in TE-enriched regions in the genome (*p*-value < 2.3 × 10^−11^) (Fig. 3C). These results revealed that chromatin accessibility in the TE-enriched regions in the ovules has a particularly great impact on gene expression. Gene Ontology (GO) analysis showed that the cluster VIII genes in the TE-enriched regions are involved in the DNA replication and nucleocytoplasmic transport (Fig. 3D). Thus, many of these genes are likely involved in the cell division during ovules, and the decreased expression of these genes during fiber elongation is due to the fact that fiber cells are arrested at the G1 stage [39, 40].

Cluster VII genes are distinctly associated with the fiber elongation stages (Fig. 3A, B). Genes in cluster VII were significantly enriched in the gene-enriched regions during the fiber elongation stage (*p*-value < 2.0 × 10^−16^) (Fig. 3C). GO analysis revealed that genes in cluster VII are involved in the Golgi vesicle transport and ER to Golgi vesicle–mediated transport (Fig. 3D), indicating that these genes may be related to the accumulation of endoplasmic reticulum (ER), Golgi body in fiber tip growing cells during fiber elongation [28].

### Correlation of RNA-seq and DNase-seq data reveals the differences in regulation between ovules and fibers

**Fig. 4:**
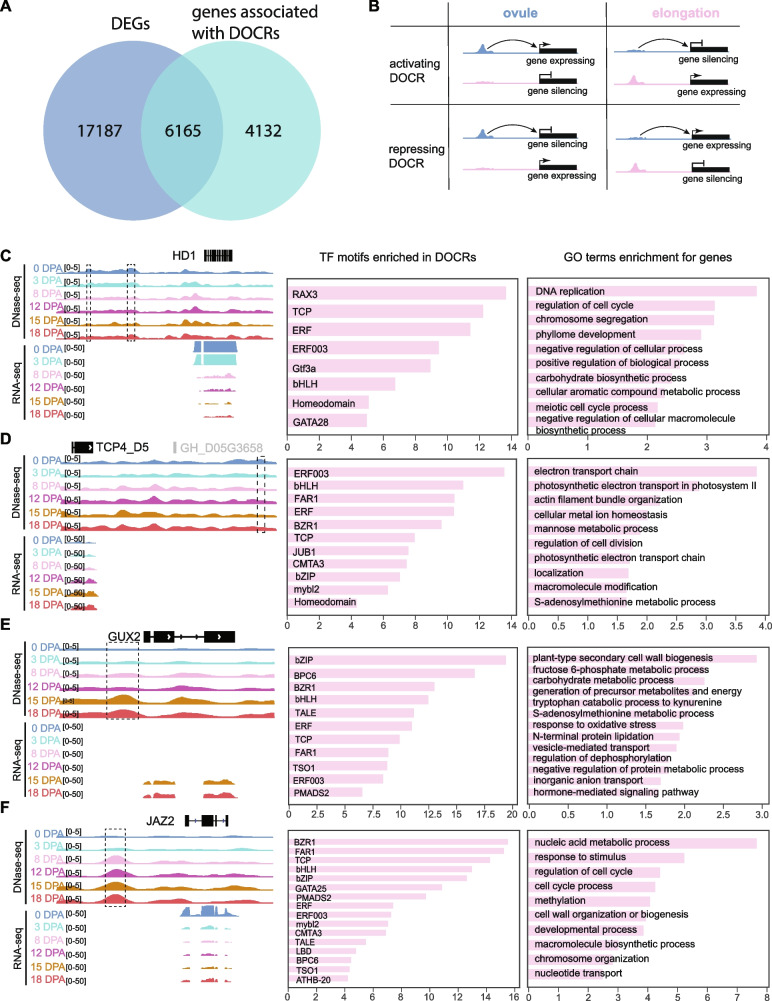
Specific enriched DNA motif associated with ovules and fiber elongation. **A** Percentage of overlap between differentially expressed genes (DEGs) and genes linked to differentially opened chromatin regions (DOCRs). **B** Schematic representation of the functional relationship between DOCRs at ovule and fiber elongation. **C–F** Left: overview of DNase-seq and RNA-seq tracks for representative ovule activating DOCRs (**C**), ovule repressing DOCRs (**D**), elongation activating DOCRs (**E**), and elongation repressing DOCRs (**F**). The dotted box represents the DOCRs. Middle: representative TF motifs enriched in activating and repressing DOCRs in the ovule and elongation domains. Right: GO terms enrichment analysis of each DOCR-related gene group was indicated. The *x*-axis represents the values of − log10(*p*-value)

To gain further insight into the differences of gene regulatory networks (GRNs) during ovule and fiber cell, we sought to identify differentially expressed genes (DEGs) and differentially open chromatin regions (DOCRs) between ovule (0 DPA) and fiber cells (8, 12, 15, and 18 DPA). To this end, a total of 23,352 DEGs and 26,373 DOCRs were identified (Fig. 4A; Additional file 5: Table S4). Interestingly, in DOCRs, the number of downregulated DOCRs (ovule-specific DOCRs) was significantly higher than that of upregulated DOCRs (elongation-specific DOCRs) (17,467 vs. 8906) (Additional file 1: Fig. S6A). We further compared the distribution differences between downregulated and upregulated DOCRs in the whole genome, and the results showed that downregulated DOCRs were significantly enriched in TE regions, while upregulated DOCRs were significantly enriched in promoter regions (Additional file 1: Fig. S6B), which showed similar distribution to DHSs during ovules and fiber elongation.

Integration of open chromatin data and gene expression data has been used to explore the architecture of developmental GRNs [41–44]. By calculating the correlation between gene expression and chromatin accessibility, we identified 14,105 unique links between DNase-seq Npeaks and genes (Additional file 6: Table S5). Among these links, 6165 (59.9%) DEGs associated with DOCRs were observed by a substantial overlap between DEGs and genes associated with DOCRs (Fig. 4A). In order to better distinguish the effect of open chromatin on gene expression, we classified DOCRs into four groups: (1) downregulated DOCRs associated with downregulated genes (ovule activating DOCRs); (2) downregulated DOCRs associated with upregulated genes (ovule repressing DOCRs); (3) upregulated DOCRs associated with upregulated genes (elongation activating DOCRs); (4) upregulated DOCRs associated with downregulated genes (elongation repressing DOCRs) (Fig. 4B). The results showed that the number of activating DOCRs and their related genes was significantly higher than that of repressing DOCRs and their related genes at ovules (chi-square test; *p-*value < 1 × 10^−16^) and showed an opposite trend during fiber elongation, suggesting the difference in the regulatory status of TFs in the two stages (chi-square test; *p-*value < 1 × 10^−8^) (Additional file 1: Fig. S6C; Additional file 7: Table S6). Although the number of ovules activating DOCRs was consistent with that of elongation activating DOCRs, the number of ovules activating DOCR-related genes was significantly less than that of elongation activating DOCR-related genes (chi-square test; *p-*value < 0.01). Illustrative examples of activating and repressing peaks are provided for the *HD1* (*GH_D06G1732*), *TCP4_D5* (*GH_D05G3657*), *GUX2* (*GH_A05G2027*), and *JAZ2* (*GH_D06G0835*) loci (Fig. 4C–F), which are all related to the fiber development [45–48].

Finally, to investigate the regulatory logic in activating and repressing DOCRs during ovules and fiber elongation, we scanned enriched motifs in each DOCRs. We found that the binding sites of the three TF families, bHLH, ERF, and TCP, were enriched in all DOCRs types, which implied the importance and universality of these three families in fiber development [49–52] (Additional file 1: Fig. S6D). Many other enriched motifs ranking higher in elongation activating DOCRs were also higher in elongation repressing regions, such as BPC6 (Protein BASIC PENTACYSTEINE6), BZR1 (brassinazole-resistant 1), bZIP, TSO1 (CRC domain-containing protein TSO1), FAR1 (Protein FAR-RED IMPAIRED RESPONSE 1), PMADS2 (Floral homeotic protein PMADS 2), and TALE binding sites (Fig. 4E, F; Additional file 1: Fig. S6D). A similar scenario was observed for ovule DOCRs, in which activating or repressing regions both showed ERF003 (ethylene-responsive transcription factor ERF003) binding sites as top enriched motifs (Fig. 4C, D). Interestingly, BZR1 binding sites were not only enriched in elongation DOCRs, but also enriched in ovule repressing DOCRs (Fig. 4E), suggesting that brassinosteroid (BR) hormone plays an important role in both ovule and elongation [53]. Similarly, ERF003 also showed enrichment in ovule DOCRs and elongation repressing DOCRs. We examined GO terms of genes linked to four regions and observed cell cycle process and DNA replication terms enriched in ovule activating DOCRs (Fig. 4C), this further suggests that the opening of chromatin in TE-enriched region during fiber ovule is related to cell division (Fig. 3). Besides, GO terms linked to cell cycle were also enriched in elongation repressing DOCRs (Fig. 4F); similarly, S-adenosylmethionine metabolic process term was enriched at both elongation activating DOCRs and ovule repressing DOCRs. Such functional overlap between ovule and elongation in activating and repressing DOCR-related genes revealed that a common set of genes are activated in the ovule and repressed in elongation by combinatorial *cis*-regulatory (Additional file 1: Fig. S6E). In addition, actin filament bundle organization terms enriched in ovule repressing DOCRs and terms associated with plant-type secondary cell wall biogenesis and hormone-mediated signaling pathway were enriched in elongation activating DOCRs (Fig. 4D, E), suggesting that the regulatory pathways related to cell elongation are inhibited during ovules and activated during elongation stage [54, 55].

### Dynamics of hormone activity during fiber development

The result above indicates showed that the binding sites of several hormone-related TF were enriched in all DOCRs, such as ERF and BZR1 (Fig. 4). This highlights that hormones play important roles in fiber development [55, 56]. We have a great interest in studying the regulatory dynamics of TFs related to hormone synthesis and response during fiber development. Therefore, we retrieved genes related to auxin, ethylene, gibberellic acid (GA), jasmonic acid (JA), cytokines (CK), brassinolide (BR), and abscisic acid (ABA) from CottonFGD [57] (Additional file 8: Table S7). To efficiently analyze the expression dynamics, we identified all expressed hormone-related genes and clustered them into different groups based on their pathways. As expected, most JA-related genes were highly expressed during 0 and 3 DPA (Additional file 1: Fig. S7A), indicating a positive regulatory role in ovules. Cytokinin contributes to the development of cotton ovule [58], and our result showed that most cytokinin-related genes were highly expressed at 0 or 3 DPA (Additional file 1: Fig. S7A). The accumulation of gibberellin is related to elongation of fibers, and its content increases rapidly after flowering, peaks at 10 DPA, and decreases rapidly thereafter [59]. Accordingly, most gibberellin-related genes were highly expressed at 0, 3, and 8 DPA, and significantly decreased at 12, 15, and 18 DPA. Auxin plays an important role in the initiation and elongation of cotton fiber cells [55]. Our result showed that there are some genes that are highly expressed at the ovules or elongation stages, for example, *GhARF2* and *GhARF18* are highly expressed at ovules and overexpression of these two genes significantly promote the initiation of trichomes in *Arabidopsis* [60]. *GhABP* is highly expressed at 8 and 12 DPA and is thought to be involved in elongation of fibrocytes [61].

We further screened hormone-related TF and compared their TF activity score in different periods. BR plays an important role in ovules and fiber elongation. *BZR1*, the core transcription factor in the BR signaling pathway, regulates the transcription of target genes by binding to BRRE boxes [62]. We found that BZR1 activity score was significantly higher at ovules than at fiber elongation stages (Additional file 1: Fig. S7B). Analysis of BZR1 downstream target genes revealed that BZR1 was involved in establishment or maintenance of microtubule cytoskeleton polarity in the ovules and involved plastid membrane, calcium ion binding, and regulation of brassinosteroid-mediated signaling pathway in elongation stage (Additional file 1: Fig. S8A). In general, in the ovules, *BZR1* may affect early fiber development by affecting microtubule establishment, and in the elongation stage, *BZR1* may affect fiber elongation by participating in signal transduction in fiber cells. Previous studies have shown that ABA inhibits fiber initiation and elongation, and the inhibition is highly related to ABA level [63]. We found that ABA-related TFs have high activity scores both at ovules and fiber elongation stages and ABA-related TFs activity scores were significantly higher at ovules than at fiber elongation stages, for example: *ABF2* and *AREB3* (Additional file 1: Fig. S7B). GO analysis revealed that ABF2 downstream target genes are involved in response to external biotic stimulus in ovules and jasmonic acid-mediated signaling pathway and plastid membrane in fiber elongation stages (Additional file 1: Fig. S8D). In cotton, ethylene synthesis is one of the most important synthetic pathways in fiber elongation, and ethylene can significantly promote elongation of fiber cells in vitro [64]. A large number of ethylene-related TFs remained highly active scores during fiber elongation. ERF3, ethylene-responsive transcription factor 3, activity score was significantly higher at fiber elongation than at ovules, which may affect fiber development by affecting microtubule polymerization or depolymerization and large ribosomal subunit pathway-related genes (Additional file 1: Fig. S8C). Auxin-related genes were highly expressed during ovules and fiber development; however, few auxin-related TFs had highly activity score at the ovules and elongation stages. In our results, ARF3, which is highly correlated with fiber quality [65], showed high TF activity score at both ovules and fiber elongation. Analysis of ARF3 downstream target genes revealed that ARF3 was involved in regulation of DNA replication in ovules and plant-type vacuole in elongation stages (Additional file 1: Fig. S8B). Overall, we described the regulatory dynamics of hormone-related TFs during ovule and fiber development from the perspective of *cis*–*trans* regulation and the regulatory pathways that TFs are involved in at different stages, adding new content to the influence of hormones on fiber development.

### Construction of a transcription factor regulatory network

**Fig. 5:**
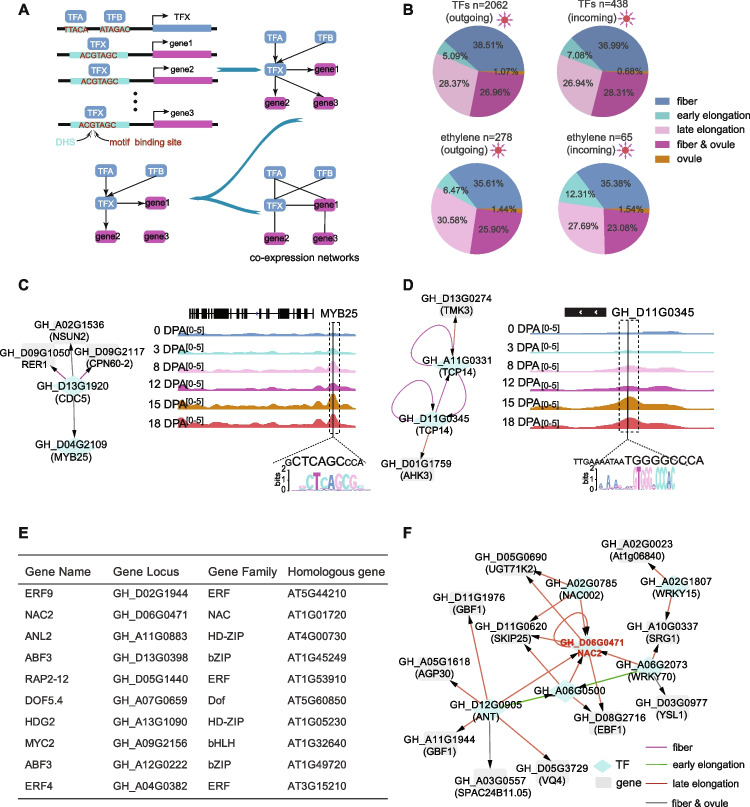
Construction of a transcription factor regulatory network. **A** To build network, an edge was created when a motif binding site of a source TF overlapped DHSs and locate in a target TF gene, including 1500 bp upstream of the target TF’s transcription start site. For example, TF A and TF B regulate TF X, while TF X controls gene 1, gene 2, and gene 3. In order to increase the credibility of the transcription factor regulatory network, the co-expressed regulatory network was generated and merged with DNase-seq network to obtain the final regulatory network. **B** Changes in regulation patterns during ovule and fiber development in outgoing and incoming connections. Outgoing connections represent the number of genes regulated by this TFs. Incoming connections represent the number of TFs regulate this TFs or genes. **C**,** D** Overview of DNase-seq tracks for representative TF interactions in cotton TF regulatory network. The TCP14 recognition motif “GTGGGNCCCAC” (large coded) was identified from the upstream DHSs of *GH_D11G0345*. The CDC5 recognition motif “CTCAGC” (large coded) was identified from the upstream DHSs of *GhMYB25*. To the left of DNase-seq tracks is the subnetwork where *GhMYB25* and *GhTCP14*. Diamonds represent TFs and rectangles represent genes. Dotted box indicates DHS regions, black line indicates CDC5 and GhTCP14 binding site. **E** Identification of hub TFs in TF network. Top 10 TFs were identified by cytoHubba. **F** Subnetworks of stress-related NAC2. Diamonds represent TFs and rectangles represent genes

Fiber growth and development is a complex regulatory process in which active TFs and CREs interact in cells. Reliable identification of specific TF-gene regulatory relationships is critical to elucidate how different biological processes such as fiber growth, development, or stress responses are transcriptionally controlled. In order to understand the regulatory mechanisms for genes related to fiber development, we constructed a TF regulatory network using DNase-seq data (Fig. 5A). In order to increase the credibility of the TF regulatory network, we first used Pearson correlation coefficients to generate a co-expression regulatory network, and then merged the two networks to obtain the final TF regulatory networks during ovules and fiber elongation (Fig. 5A; Additional file 9: Table S8).

We next examined the interactions in the TF regulatory networks. A total of 503 TFs were found to interact with each other in the networks, including a similar number of TFs derived from the At subgenome genes (248) and the Dt subgenome genes (255). The TF networks allow us to reveal the interaction between members of different TF families, which helps us to understand the roles of TF families in the ovules and fiber elongation stages. Five hub families, C2H2, bZIP, WRKY, ERF, and bHLH, were identified in the network (Additional file 1: Fig. S9A). GO analysis revealed different contributions of these family-related genes to fiber development. Members of the bZIP family are mainly involved in positive regulation of abscisic acid-activated signaling pathway and response to alcohol; C2H2 family members are mainly involved in asymmetric cell division; ERF family members regulate the ethylene-activated signaling pathway; WRKY family members are involved in cellular response to boron-containing substance deprivation; bHLH family members are involved in the positive regulation of flavonoid biosynthetic process (Additional file 1: Fig. S9B).

To further understand the dynamics in regulatory networks during ovule and fiber development, we divide regulatory relationships in networks into 5 categories: (1) ovule (connect only at 0 or 3 DPA); (2) fiber and ovule (connect at ovule and fiber elongation (12, 15, and 18 DPA)); (3) fiber (connect at 8 and 12, 15, or 18 DPA); (4) early elongation (connect at 8 and not at 12, 15, and 18 DPA); (5) late elongation (connect only at 12, 15, or 18 DPA). The results showed that the regulation was the most abundant in the fiber development period, especially in the late elongation, while the specific regulation in the ovule period only accounted for 1.07% (Fig. 5B). We also focused on changes in the regulation of hormone-related genes during fiber development. In the network, the outgoing connection shows that ethylene, ABA, auxin, and BR play an important role in ovule and fiber development (Fig. 5B; Additional file 1: Fig. S9C-9G). Interestingly, a greater proportion of incoming connections in ethylene, auxin, and BR occur in early elongation than outgoing connections. In BR, however, incoming connections tend to extend late elongation. These results indicate that hormone-related factors have significant tissue specific in the relationship between outgoing connection and incoming connection. We found that there is a subnetwork enriched with ethylene-related factors and the regulation dynamics of ethylene-related genes during fiber development were clearly demonstrated (Additional file 1: Fig. S10). ERF4 (*GH_D05G3685*) is highly expressed during ovule and fiber development (FPKM of ovule 86.5; FPKM of fiber 32.1). However, this transcription factor has only high activity during fiber development, and downstream regulates GATA5, REFA1, and other genes. Interestingly, ERF4 is regulated by other transcription factors, such as ERF017 and GATA5, in the early elongation. In addition, ERF9 (*GH_A03G1781*) is highly expressed during ovule and fiber development (FPKM of ovule 31.7; FPKM of fiber 12.5), and it has multiple regulatory genes throughout development.

### Revealing roles of TFs in fiber development through regulatory networks

The interaction model of TF regulatory network revealed the regulation of several known genes related to fiber development. *GhMYB25* was identified to play an important role in fiber initiation and elongation. *GhMYB25*-silenced cotton showed shorter fibers and a sharp decrease in trichomes in other parts of the plant. Ectopic overexpression of *GhMYB25* resulted in an increase in cotton fiber initiation and leaf trichome number [66]. Interestingly, our network shows that *GhMYB25* is regulated by CDC5 (*GH_D13G1920*) during ovule and fiber development, a cell division cycle 5-like protein (Fig. 5C). These results suggest that CDC5 may regulate the expression of *GhMYB25* and thus contribute to the number of fibrocytes. *GhTCP14* (*GH_A11G0331*) is transcribed at high levels during fiber initiation and elongation. *GhTCP14* may play an important role in the regulation of auxin-mediated differentiation and elongation of cotton fiber cells [50]. *GH_D11G0345* is the homoeologous gene of *GhTCP14*, which is highly expressed during ovules and fiber elongation stages, and is believed to affect fiber development [67]. In the TF regulatory network, these two genes are both self-regulated and mutually regulated during fiber development (Fig. 5D). In addition, we also found contextual regulation of *GhHOX3* (*GH_A05G1312*), *GhSLR1* (*GH_A07G0869*), and *GhBZR1* (*GH_A05G1959*) in the regulatory network, which is known TF related to fiber development (Additional file 9: Table S8).

We further screened 10 hub genes through regulatory network (Fig. 5E, F; Additional file 1: Fig. S10 and S11). Many of these hub genes are related to hormones. Examples include ethylene response factors (ERF9, ERF4, and RAP2-12), and abscisic acid insensitivity factors (ABF3). In addition, MYC2 can interact with JAZ and EIN3 to mediate the gibberellin and ethylene crosstalk [68]. In *Arabidopsis*, *Dof5.4* (Dof zinc finger protein DOF5.4) is a regulator of root growth and cell elongation and differentiation [69]. The *HDG2* gene is required for normal gl3-sst sim trichome development [70]. During the fiber initiation and elongation stages, fiber cells accumulate a large amount of solute to maintain the required turgor pressure. The expression of stress response factors is similar to stress-like conditions being generated due to turgor pressure. In the *fl* mutant, the expression of TF genes from the AP2-EREBP, C2H2, NAC, and WRKY families were significantly downregulated at 0 and 5 DPA [71]. These genes involved in stress responses and cellular respiration are related to fiber cell wall thickness [72]. Therefore, there are stress-related genes in the TF regulatory network, which contribute to fiber development. There are many phytohormones with primary functions in biotic and abiotic stress responses, for example: ERF4, ERF9, and ABF3 all respond to abiotic stress in *Arabidopsis* [73, 74]. In addition, *GhNAC2* plays a positive role in drought resistance of cotton by regulating the expression of GhNCED3a/3c, ABA biosynthesis, and stomatal closure [75]. Overall, our regulatory network analysis illustrates the regulation of fiber-related TFs during fiber development and predicts some important factors during fiber development.

### Characterization of a new TF related to fiber development

**Fig. 6:**
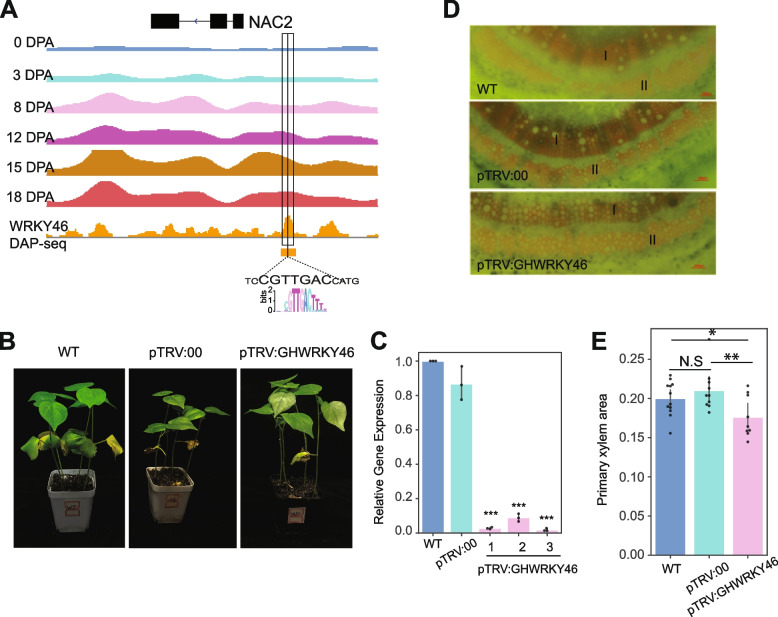
Roles of TFs in fiber development revealed through regulatory networks. **A** *GhNAC2* (*GH_D06G0471*) interact with Gh*WRKY46* (*GH_A06G2073*). The DNase-seq tracks for the *GhNAC2* loci are shown. The WRKY46 recognition motif “CNTTGAC” (large coded) was identified from the upstream DHSs of *GhNAC2*. Orange is DAP-seq and peaks. Shade boxes indicates DHS region. **B** The phenotypes of plants inoculated with pTRV:00 and pTRV:*GhWRKY46*. The pTRV:00 vector was used as a vector control. **C** Relative transcript levels of *GhWRKY46* in systemic leaves of cotton infiltrated with pTRV:00 and pTRV:*GhWRKY46*. The WT value was set as 1. Data are shown as mean values ± SD (*n* = 3). (Student’s *t* test, *** *p* < 0.001). **D** Cross-cut lignin histochemical staining analysis of cotton stalks. I: Primary xylem; II: Primary phloem. Scale bar, 100 µm. **E** Primary xylem area proportion in systemic stem of cotton infiltrated with pTRV:00 and pTRV:*GhWRKY46*. (Student’s *t* test, * *p* < 0.05, ** *p* < 0.01, N.S, not significant, *n* = 12, 10, 9, respectively)

In *Gossypium raimondii*, the expression of *WRKY46* was upregulated after drought treatment and salt treatment [76]. Our data showed that in *Gossypium hirsutum*, gene *GhWRKY46* (*GH_A06G2073*) expressed in both ovule and fiber elongation. Interestingly, in the TF regulatory network, GhWRKY46 regulates a downstream gene *GhNAC2* (Fig. 5F). DAP-seq (DNA affinity purification sequencing) uses in vitro-expressed TF to obtain TF-bound DNA fragments in the genome and to establish TF binding sites and binding motifs [77]. In *Arabidopsis thaliana*, DAP-seq combing previously identified DHSs established TF-binding sites with high accuracy [78]. In order to further validate the interaction between *GhWRKY46* and *GhNAC2*, we conducted a DAP-seq experiment on *GhWRKY46*. We obtained a total of 21,931 DAP peaks from the DAP-seq libraries, and these peaks were enriched in the GTTGACTTT motif, which was consistent with the protein-binding microarrays (PBM) (Additional file 1: Fig. S12A) [78]. In addition, the DAP peaks overlapped with DHSs that were significantly enriched in PBM binding sites (*p*-value < 1.0 × 10^−11^; hypergeometric test), which is consistent with the enrichment of motifs in the open chromatin regions (Additional file 1: Fig. S12B). The DAP peaks contain a total of 4931 (22.5%) binding sites of PBM as predicted by FIMO software, which are considered to be WRKY46 binding sites. We first analyzed whether the WRKY46 binding sites had regulatory preference during fiber development. We calculated the overlap ratio between the DHSs and WRKY46 binding sites at each stage; ~ 2.6–9.0% of WRKY46 binding sites overlapped with open chromatin at fiber initiation and elongation. Also, the enrichment of WRKY46 binding sites in the fiber elongation stage was similar to that at the initiation stage (Additional file 1: Fig. S12C). As expected, a WRKY46 binding site was identified in the *GhNAC2* promoter region (Fig. 6A), indicating that *GhNAC2* is indeed a downstream gene of *GhWRKY46*. We also found that two TFs encoded by *GH_A02G0785* and *GH_A06G0500* likely participate in the regulation of *GhNAC2*. To further investigate the function of *GhWRKY46*, we used virus-induced gene silencing (VIGS) to silence the gene. When we used phloroglucinol to analyze the changes in xylem content in the silenced plants, which is the main component of cell wall synthesis, the results showed that the proportion of primary xylem area decreased significantly in the gene-silenced seedlings (Fig. 6B–E). These results suggest that WRKY46 is likely involved in cell wall synthesis during fiber development. In general, we established a reliable transcription factor regulatory network, which provides a reference for future study of the transcriptional regulation of genes related to fiber development.

## Discussion

Extensive genomic and functional genomic studies have been conducted in cotton. However, there have been only few studies related to analysis of regulatory dynamics, especially during cotton fiber development. Here, we constructed genome-wide chromatin accessibility maps using DNase-seq, RNA-seq, and ChIP-seq during cotton ovules and fiber elongation stages. Our data revealed an open chromatin dynamic in these development stages and offered a global view of the transcriptional regulatory elements related to fiber development. Our results also provide a valuable resource for elucidation of the transcriptional networks that control fiber development.

In this study, we developed and sequenced a DNase-seq using a single-end 50 bp strategy that generates ~ 20 bp sequence reads [13]. Ninety-six percent of DNase-seq reads could be mapped to the cotton genome; however, only ~ 30% of DNase-seq reads were mapped to unique positions. The reason for this phenomenon is that the cotton genome contains a large number of repeat sequences, and the ~ 20 bp reads are too short, resulting in sequences mapped to multiple locations. In contrast, the short length of sequence reads is not a problem for genomes with less repetitive sequences, such as Arabidopsis (~ 70% unique mapping reads) [12]. As technology continues to evolve, paired-end long-read sequencing has emerged as a more suitable approach for open chromatin analysis of genome with high-repetitive sequences, such as ATAC-seq and modified DNase-seq method (mDNase-seq) [16, 79].

Previous study on DNA methylation and chromatin dynamics in cotton revealed that chromatin accessibility decreased in pericentromeric regions during fiber development (0, 10, 20, and 30 DPA) [26]. In our study, we constructed genome-wide DHS maps for cotton ovules and fiber elongation. We found that the number of DHSs at the ovule stage was significantly lower than that at the fiber stage. Recently, Pei et al. revealed that histone modifications during the ovule period are less than those during the fiber period [27]. From ovule to fiber, we demonstrate that the chromatin accessibility during fiber development increased gradually in the gene-enriched regions but decreased gradually in the TE-enriched regions, which is consistent with the previous report [26]. Previous studies have shown that fibrocytes have additional CHH hypermethylation in the heterochromatin region compared to ovules, resulting in the silencing of TE and nearby genes [25]. It has been shown that CG and non-CG methylation promote chromatin inaccessibility [80]. These results suggest that epigenetic modifications undergo drastic remodeling during ovule-to-fiber development. Termination of the maternal program and activation of the syngeneic genome are key processes in plant and animal embryogenesis [81–83]. During this process, epigenetic modifications are reprogrammed. As well, the chromatin gradually gained accessibility during zygotic activation process in both human [84] and wheat [85]. In plants, fertilization and further embryo development occur within the ovule. In this study, 0 and 3 DPA materials were derived from ovules whose chromatin state was more inclined to early embryonic development. Fibers are differentiated from ovule epidermal cells [86]. The cell states during the fiber period represent mature, differentiating cells. Therefore, we suggest that histone modifications and chromatin accessibility undergo dramatic remodeling during ovule-to-fiber development to establish a favorable chromatin environment to promote fiber elongation. As expected, the specifically expressed affected genes by open chromatin in fibers may affect fiber elongation through fiber tip growth cells.

TE is an important component of regulatory elements. Pei et al. uncovered active TE plays a topological role in demarcating TAD-like structure in cotton fiber, indicating that it may be involved in chromatin interaction [27]. Here, we identified a large number of teDHSs in ovules and fibers and confirmed their enhancer function, which also implies that TE may be involved in chromatin interactions. To gain further insight into the differences of gene regulatory networks (GRNs) during ovule and fiber cells, we used proximity methods to correlate genes with OCRs, which have been widely used [41–44]. We identified 26,373 differentially open chromatin regions (DOCRs) between ovule and fiber cells and 14,105 unique links between DNase-seq peaks and genes. We described in detail the characterization of DOCRs and related DEGs that are activated and repressed during ovules and fiber elongation. We have shown that both ovule activating DOCR-related genes and elongation repressing DOCR-related genes are involved in cell cycle and DNA replication pathway, indicating that they are antagonistically regulating a similar set of genes.

In recent decades, an increasing number of genes and pathways related to cotton fiber development have been identified [23, 87–94]. Despite the discovery of these key regulators, the architecture of the fiber development regulatory networks is far from fully understood. Regulatory networks are powerful tools for the study of genome-wide transcriptional regulation [95]. We therefore constructed a TF regulatory network to tease out the connections among the relevant TFs during fiber development. We combined sequence-specific TF networks and co-expression networks to obtain a final comprehensive network. We found some known regulatory relationships within the network. For example, in *Arabidopsis*, MYC2 (*AT1G32640*) self-inhibit its own expression [96], and ORC2 (*AT2G37560*) is a target gene of the E2F family [97]. In our network, *GH_D09G2092* (*GhMYC2*) exhibits autoregulatory loop, and E2FA (*GH_D01G1470*) regulates *ORC2* (*GH_A11G3718*) expression by binding to the *ORC2* promoter. In addition, DAP-seq combined with other sequencing data has been used to determine the binding sites of transcription factors [78, 98]. Based on the DAP-seq, we confirmed the interactions between *GhBES1.4* and *GhSAD2* [98], as well as novel interactions between *GhWRKY46* and *GhNAC2*. Overall, these results collectively confirm the reliability of our regulatory network. The analysis of regulatory networks revealed the contextual regulation of some known fiber development-related factors and allowed us to predict and characterize some hub genes. Hormones and stress response factors play important roles in fiber development. In network, we found interactions associated with stress-related genes, such as the interactions between *GhWRKY46* (*GH_A06G2073*) and *GhNAC2* (*GH_D06G0471*) [75, 76]. We found a significant reduction in lignin content in plants with VIGS-silenced *GhWRKY46* genes, and the results suggest that it may participate in fiber growth and development by regulating lignin content. Moreover, the data from our study will facilitate future functional genomics studies in cotton and will help inform breeding strategies.

## Conclusions

The utilization of *cis*-regulation in fiber development stages is a new direction of cotton molecular breeding. This work reveals a dynamic chromatin accessibility in ovules and fiber development stages and a large number of TEs act as enhancers to affect DNA replication during ovule. This provides a new insight for understanding the regulatory landscape of cotton genome. The discovery of new genes related to fiber development has always been a priority for cotton breeding, and the efficient and reliable large-scale prediction of genes related to fiber development is very important. In this study, using DNase-Seq and RNA-seq data, a high-confidence regulatory network for ovule and fiber elongation was constructed. According to the regulatory network, we predicted important transcription factors during fiber development and experimentally verified the interaction of the network and identified novel fiber development-related genes. Overall, our analysis results will benefit the research of cotton molecular breeding.

## Methods

### Plant materials

Upland cotton (*Gossypium hirsutum* L. TM-1) plants were cultivated in a field in Anyang, China, and were managed regularly during the summer from April 25 to October 25, 2018. At the 0 DPA ovule stage, we collected the flowers to detach the ovule (0 DPA), the cotton boll wall was eliminated by tweezer to expose ovules, the ovules were detached and immediately frozen in liquid nitrogen, and the ovules from different bolls were collected together as one sample. After 3 days from marked date, we collected samples for 3 DPA according to the 0 DPA sample collection method. After 8 days from marked date, we collected the marked cotton bolls, and then the cotton boll wall was eliminated by tweezer to expose fiber, the fiber was eliminated by tweezer from the ovules, and the fiber from different bolls were collected together as one sample. The samples for 12, 15, and 18 DPA were collected according to the 8 DPA sample collection method. Samples from different plants were pooled and immediately frozen in liquid nitrogen and stored at − 80℃ until further use.

### DNase-seq, ChIP-seq, and RNA-seq

The DNase-seq experiments were performed as previously described [99], except that in the nuclei isolation steps, 2% PVP 40 (Solarbio, catalog number 822B047) was added to the nuclei isolation buffer and nuclei washing buffer to extract high-quality nuclei. DNase-seq libraries were constructed with two biological replicates for each sample and sequenced on the Illumina platform to generate 50-bp single-ended reads [99].

Chromatin immunoprecipitation (ChIP) was performed using published protocols with minor modifications [100]. As above, 2% PVP40 was added to the nuclei isolation buffer and nuclei washing buffer. Two commercial antibodies directed against H3K4me3 (abcam ab8580) and H3K27me3 (Millipore 07–449) were used to obtain immunoprecipitated DNA. ChIP-seq libraries were constructed as previously described, and sequenced using the paired-end 150 base pairs (PE150) strategy [13, 100].

Total RNA for each sample was extracted using TRIzol reagent and sequenced on an Illumina HiSeq sequencing system. Six biological replicates were developed for RNA-seq for each sample.

### DNase-seq analysis

We performed DNase-seq on DNA from 0 and 3 DPA at ovules and developing cotton fibers at 8, 12, 15, and 18 DPA, and obtained a total of 2.216 billion sequence reads. The reads were aligned to the upland cotton genome [101] using bowtie (-m 1 -n 1) [102]. To confirm the reproducibility and reliability of DNase-seq, we calculated the Pearson correlation coefficients and the rescue ratio between the biological replicates of the data from the same developmental stage. The cotton genome was divided into 5-kb non-overlapping windows. The number of DNase-seq reads in the windows was used to calculate the Pearson correlation between replicates. The rescue ratio is used to measure consistency between biological replications, while the self-consistency ratio is used to measure consistency within a single dataset [103] (https://www.encodeproject.org/data-standards/terms/). The rescue ratio and self-consistency ratio were calculated according to protocols (https://github.com/cejuliano/jcazet_regeneration_patterning), with slight modifications. Briefly, for rescue ratio, we used samtools to generate pseudo-replicates of the merged replicates. Each replicate DHS was identified by F-seq2 [104] with the parameter “-tp 4 -f 0.” We used IDR (Irreproducible Discovery Rate) to calculate the number of peaks passing the determined IDR threshold (0.1) and to calculate the ratio of the number between true and re-sampled pseudo-replicates. The Pearson correlation coefficients ranged from 0.95 to 0.99 and the rescue ratio ranged from 0.71 to 0.95, indicating that the data sets are highly reproducible and that the identified DHSs are indeed reliable [29] (Additional file 2: Table S1). DHSs were identified using F-seq2 with the parameter “-tp 6 -p 0.01 -f 0.” We used IDR to get consistent peaks between replicates [105]. We used BEDtools intersect with parameter “-f 0.5” to annotate the DHSs relative to genes [106]. We used BEDtools intersect with parameter “-f 1” to annotate the motifs relative to DHSs. TE-derived DHSs (teDHSs) were identified by determining the genomic coordinates of DHSs overlapping by at least 30% using the BEDtools intersect. We used R package UpSetR to draw upset plots [107].

### RNA-seq analysis and ChIP-seq analysis

We conducted a series of RNA-seq and ChIP-seq using the same fiber tissues that were used for DNase-seq. The reads from RNA-seq and ChIP-seq were aligned to the upland cotton genome [101] using hisat2 [108] and bowtie2 v2.3.5 [109], respectively. The gene expression levels were calculated using StringTie [110] with the default parameters. There were six biological replicates of RNA-seq for each tissue that were highly correlated (Pearson’s rank correlation coefficients [SC] ranged from 0.94 to 0.99) (Additional file 1: Fig. S13).

### Motif finding, motif scanning, and enrichment testing

We downloaded the *Gossypium hirsutum* TF genes with well-annotated recognition motifs from the plantTFDB database [111] and the biomart database [112]. We obtained a total of 757 motifs and assigned them to 2408 transcription factors (Additional file 10: Table S9). We used FIMO [113] with the default parameters to scan for motifs. We calculated the enrichment of each motif using hypergeometric distribution (*p*-value < 0.001).

### Identification of differentially expressed genes (DEGs) and differentially open chromatin regions (DOCRs)

DEseq2 was used to identified DEGs and DOCRs. Specifically, we first compared the differential expressions elongation periods (8, 12, 15, and 18 DPA) and 0 DPA respectively. All DEGs with an adjusted *p*-value < 0.01 and |log_2_foldchange|> 1. When at least two elongation periods were differentially upregulated or downregulated, we considered this gene to be differentially expressed during ovules and fiber elongation. We identified the DOCRs using the same method as above. All DOCRs with a *p*-value < 0.05 and |log_2_foldchange|> 1.

The putative causal links between DNase-seq peaks and gene expression was identified by correlation-based approach. First, all possible interactions between DOCRs and DEGs within 50 kb were identified. For each peak-gene pair, we computed the observed Pearson correlation between DOCR accessibility (DEseq2 normalized count) and the gene’s expression (DEseq2 normalized count). To estimate the background, we correlated the 10,000 random peaks (within 50 kb of a gene body) accessibility to the expression of every gene. We calculated the mean and standard deviation for these random peak correlations to represent expected correlations. The *Z* score is calculated by.

$$z\mathit\;\mathit=\frac{observed\mathit\;\mathit-\mathit\;expected\mathit.mean}{expected\mathit.std}.$$

And we used a one-sided *z*-test to determine *p*-values. We then selected all correlations with a *p*-value < 0.05.

### Calculation of co-expressed genes

The co-expressed genes were calculated according to protocols described previously [114]. Briefly, we first calculated the Pearson correlation coefficients (PCC) between two genes among the 36 samples. The closer the relationship between the genes, the higher the PCC score. MR, an algorithm for calculating the PCC rank, takes a geometric average of the PCC rank from gene A to gene B and from gene B to gene A. We used PCC and MR to construct a gene co-expression network. Here, we retained co-expressed gene pairs with a single direction PCC rank (RankAB or RankBA) of < 3 and an MR score < 50 in the co-expression network, and these gene pairs were regarded as having positive co-expression relationships when their PCC values were > 0 and negative co-expression relationships when their values were < 0.

### Network building

First, we constructed a TF regulatory network using DNase-seq data. To build network, we used FIMO [113] with the default parameters to scan for motifs, and an edge was created when a motif binding site of a source TF overlapped with the DHS of the target gene (DHS locate in a 1500 bp upstream of gene’s transcription start site). In order to increase the credibility of the transcription factor regulatory network, the co-expressed regulatory network was generated and merged with DNase-seq network to obtain the final regulatory network. For example, if TF X controls gene 1, gene 2, and gene 3 in DNase-seq, and TF X is co-expressed with gene 1 and gene 2, then we consider TF X as a regulator of gene 1 and gene 2.

### DAP data analysis

Raw sequencing reads were trimmed to remove adapters using skewer [115]. Trimmed reads were mapped to the cotton reference genome using bowtie2 v2.3.5 [109]. We selected the unique mapped reads for further analysis. Peaks were called using GEM v3.4 [116] using the GST-HALO negative control sample for background subtraction. Peak calling was performed using the main parameters: –k_min 6 –k_max 13 –fold 2 –outBED.

## Supplementary Information

**Additional file 1: Figure S1.** Motifs were significantly enriched in teDHSs. **Figure S2.** RFP fluorescence observed by confocal microscopy of teDHSs. **Figure S3.** Genome wide mapping of chromatin accessibility and histone modification in cotton. **Figure S4.** Distribution changes of DNase-seq, H3K4me3 and H3K27me3 along Chromosome between 0 DPA and 8 DPA. **Figure S5.** Relative transcript level and promoter DHSs changes for cluster I-VIII genes. **Figure S6.** DOCRs configuration in the initiation and elongation. **Figure S7.** Dynamic of hormone activity during fiber development. **Figure S8.** Gene Ontology (GO) terms with BZR1, ARF3, ERF3 and ABF2 downstream target genes, respectively. **Figure S9.** Dynamics in transcription factor regulation in regulatory networks. **Figure S10 and S11.** Subnetworks of hub TFs. **Figure S12.** The relationship between DAP-seq and DHSs of fiber development stage. **Figure S13.** RNA-seq data correlation among different biological replicates and different technical replicates.

**Additional file 2: Table S1.** Summary of DNase-seq data sets.

**Additional file 3: Table S2.** Summary of functional validation of DHSs using a fluorescence reporting assay.

**Additional file 4: Table S3.** Summary of gene expression and epigenetic modification in centromere regions.

**Additional file 5: Table S4.** Summary of DEGs and DOCRs.

**Additional file 6: Table S5.** Summary of unique links between DNase-seq peaks and genes.

**Additional file 7: Table S6.** Summary of activated and repressed DOCRs and its related DEGs at ovule and fiber elongation.

**Additional file 8: Table S7.** Summary of hormones related genes from cottonfgd.

**Additional file 9: Table S8.** TF regulatory networks during fiber development.

**Additional file 10: Table S9.** Cotton TFs and their corresponding motifs.

## Data Availability

All datasets have been deposited to Beijing Institute of Genomics Data Center (http://bigd.big.ac.cn) under BioProject PRJCA006537 [117] and the National Center for Biotechnology Information databases under the accession number PRJNA719256 [118]. The processed data can be visualized in a genome browser (https://zhangtaolab.org/cottondhs) [119].

## Abbreviations

DHS: DNase I hypersensitive site
DPA: Days post anthesis
TE: Transposable elements
TF: Transcription factor
ACR: Accessible chromatin regions
teDHSs: Transposable element (TE)-derived DHSs
TSS: Transcription termination sites
TIR: Terminal inverted repeat
LTR: Long terminal repeat
GO: Gene Ontology
GRNs: Gene regulatory networks
DEGs: Differentially expressed genes
DOCRs: Differentially open chromatin regions
GA: Gibberellic acid
JA: Jasmonic acid
CK: Cytokines
BR: Brassinolide
ABA: Abscisic acid
PBM: Protein binding microarrays
VIGS: Virus-induced gene silencing
PCC: Pearson correlation coefficients
