# Supplementary material for: Genome-wide chromatin accessibility landscape and dynamics of transcription factor networks during ovule and fiber development in cotton
Source: BMC Biol. 2023 Jul 31;21:165. doi: 10.1186/s12915-023-01665-4 (PMC10391996; doi:10.1186/s12915-023-01665-4)
Supplement: Supplementary file 1 — Additional file 1: Figure S1. Motifs were significantly enriched in teDHSs. Figure S2. RFP fluorescence observed by confocal microscopy of teDHSs. Figure S3. Genome wide mapping of chromatin accessibility and histone modification in cotton. Figure S4. Distribution changes of DNase-seq, H3K4me3 and H3K27me3 along Chromosome between 0 DPA and 8 DPA. Figure S5. Relative transcript level and promoter DHSs changes for cluster I-VIII genes. Figure S6. DOCRs configuration in the initiation and elongation. Figure S7. Dynamic of hormone activity during fiber development. Figure S8. Gene Ontology (GO) terms with BZR1, ARF3, ERF3 and ABF2 downstream target genes, respectively. Figure S9. Dynamics in transcription factor regulation in regulatory networks. Figure S10 and S11. Subnetworks of hub TFs. Figure S12. The relationship between DAP-seq and DHSs of fiber development stage. Figure S13. RNA-seq data correlation among different biological replicates and different technical replicates. [file 12915_2023_1665_MOESM1_ESM.pdf]

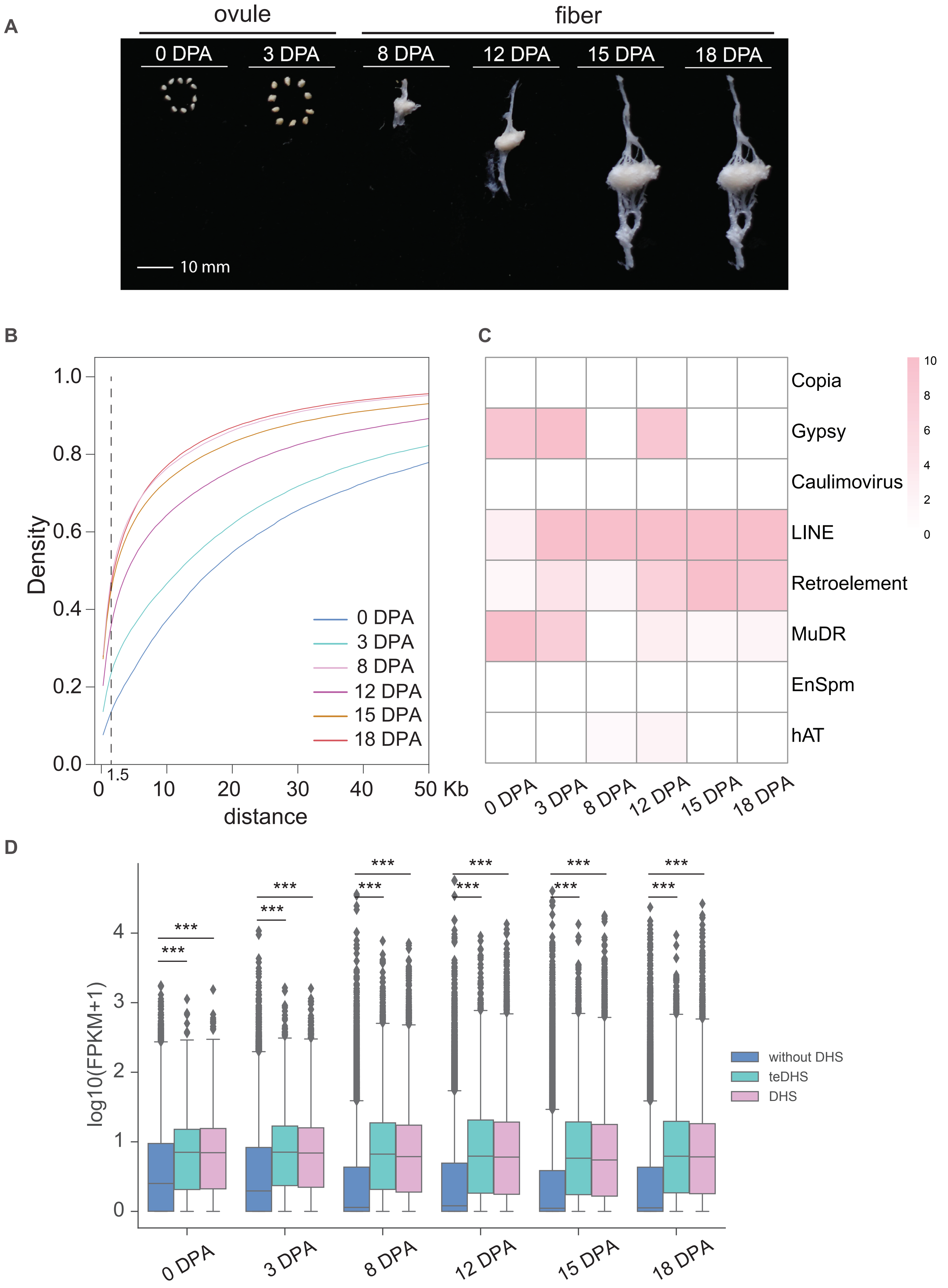

A
ovule
fiber
0 DPA
3 DPA
8 DPA
12 DPA
15 DPA
18 DPA
10 mm
B
Density
1.0
0.8
0.6
0.4
0.2
0.0
0
1.5
10
20
30
40
50 Kb
distance
0 DPA
3 DPA
8 DPA
12 DPA
15 DPA
18 DPA
C
Copia
Gypsy
Caulimovirus
LINE
Retroelement
MuDR
EnSpm
hAT
10
8
6
4
2
0
0 DPA
3 DPA
8 DPA
12 DPA
15 DPA
18 DPA
D
log10(FPKM+1)
4
3
2
1
0
***
without DHS
teDHS
DHS
0 DPA
3 DPA
8 DPA
12 DPA
15 DPA
18 DPA

Figure S1. Motifs were significantly enriched in teDHSs.

(**A**) Samples used for genome-wide DNase-seq, ChIP-seq and RNA-seq. The samples collected from field include ovules at 0 DPA and 3 DPA and fibres at 8 DPA, 12 DPA, 15 DPA and 18 DPA.

(**B**) Cumulative analysis of the distance from a teDHSs to the closest gene. The y-axis represents the proportions of teDHSs within cumulative distances.

(C) Contributions of different TE families to teDHSs during fiber development. The heat map shows the value of $-\log_{10}(p\text{-value})$. The *p*-value between observed and expected proportions was calculated by hypergeometric distribution.

(D) Compare the expression levels between genes with teDHS and without DHS. Statistical significances were calculated by Kolmogorov-Smirnov (K-S) test (***p<0.001).

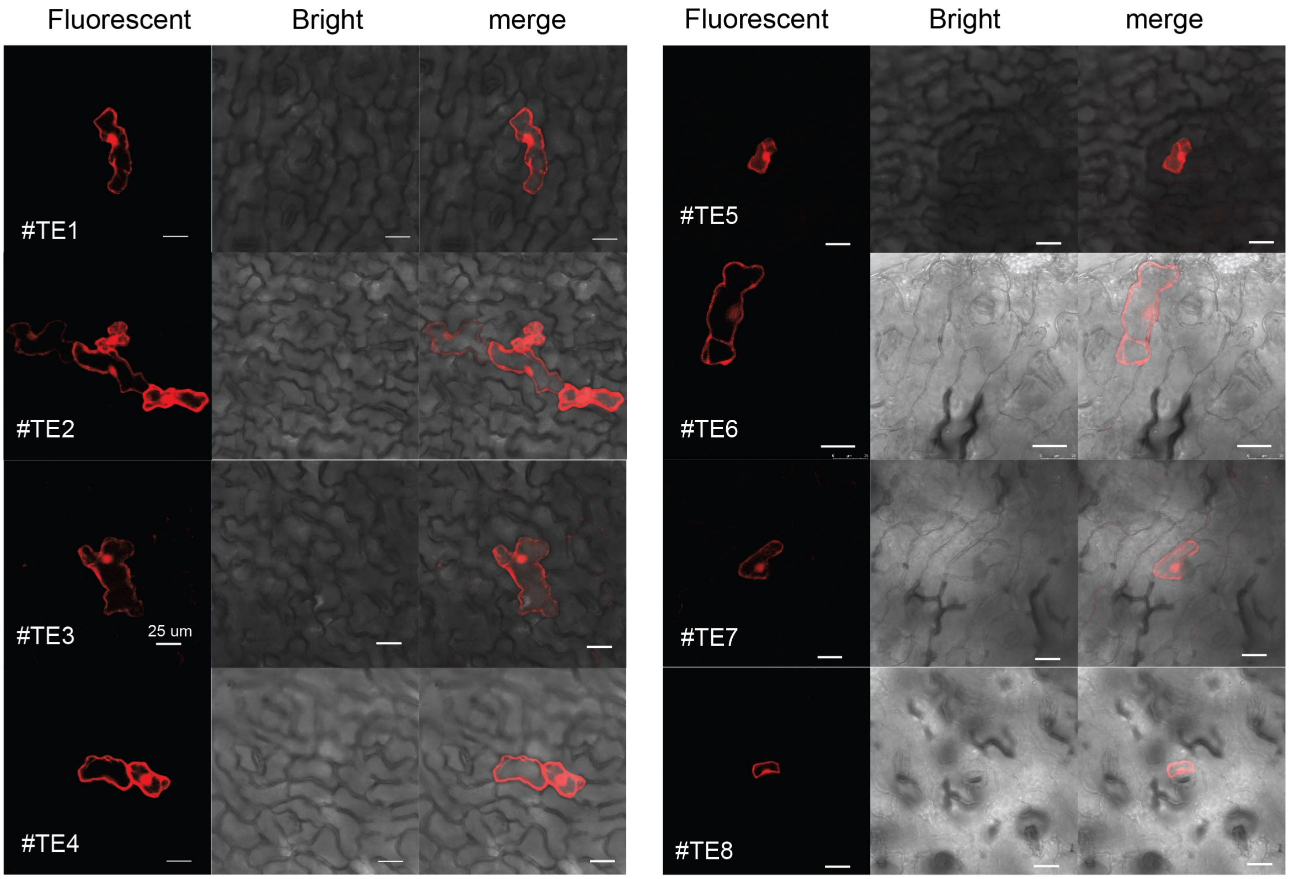

Figure S2. RFP fluorescence observed by confocal microscopy of teDHSs.

Fluorescent, bright-field and merge (bright-field and red fluorescent) confocal laser scanning micrographs of cotton leaf cells bombarded with teDHSs. 8 activate teDHSs are showing. Scale bar, 25 μm.

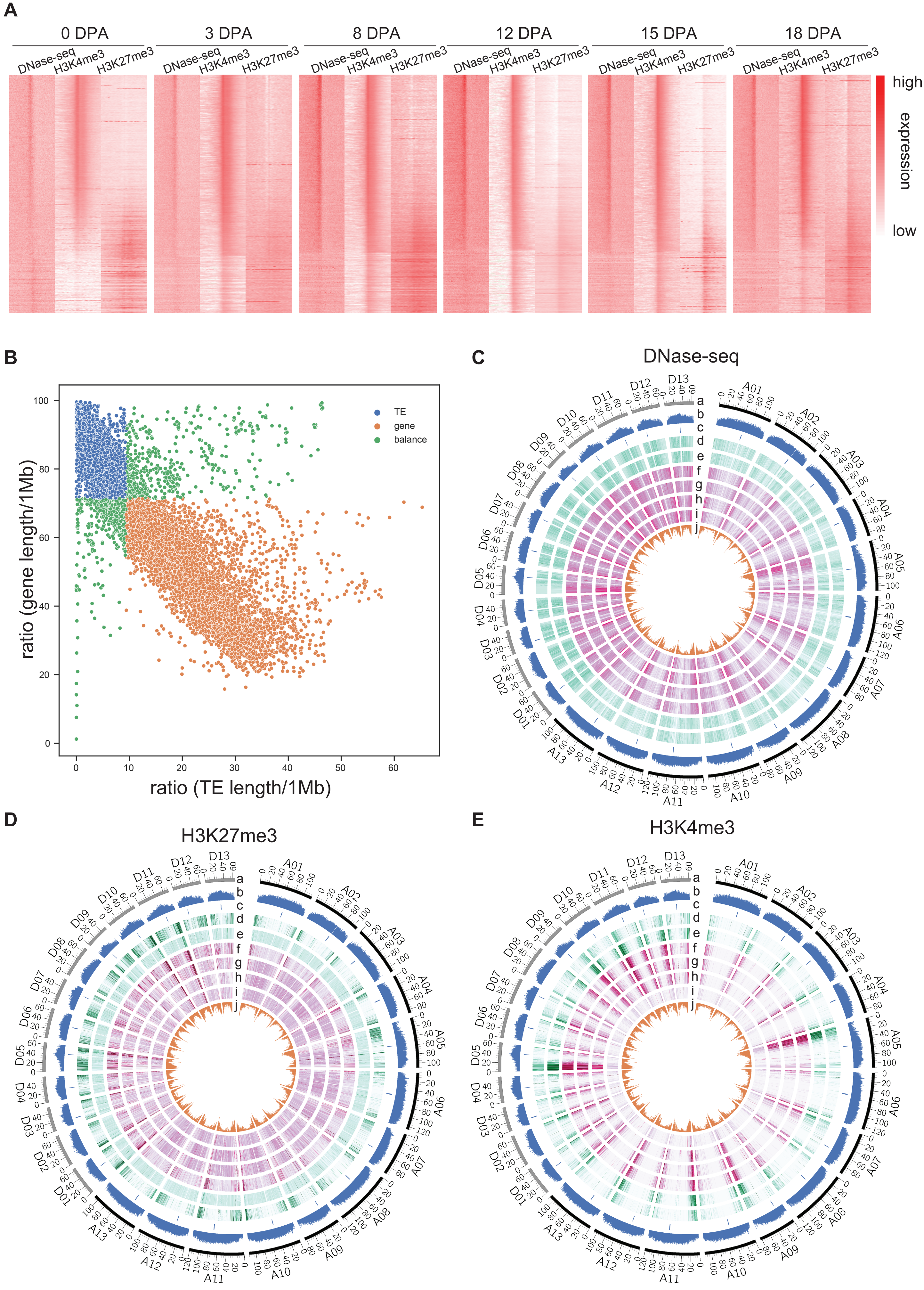
A
0 DPA
3 DPA
8 DPA
12 DPA
15 DPA
18 DPA
DNase-seq
H3K4me3
H3K27me3
high
expression
low
B
TE
gene
balance
ratio (gene length/1Mb)
ratio (TE length/1Mb)
C
DNase-seq
D
H3K27me3
E
H3K4me3
a
b
c
d
e
f
g
h
i
j
A01
A02
A03
A04
A05
A06
A07
A08
A09
A10
A11
A12
A13
D01
D02
D03
D04
D05
D06
D07
D08
D09
D10
D11
D12
D13

Figure S3. Genome-wide mapping of chromatin accessibility and histone modification in cotton.

(A) Heatmaps of DNase-seq, H3K4me3 and H3K27me3 around the TSS region (±1.5 Kb) of genes at fiber development. All genes were sorted by the expression levels (FPKM).

(B) Identification of gene and TE enriched regions in chromosome. Calculate the proportional coverage between genes and TEs in 1Mb windows 10 Kb step.

(C, D and E) Genome-wide visualization of DNase-seq reads density(C), H3K27me3 reads density (D), H3K4me3 reads density (E) in cotton and aligned against other genomic features. (a): the ideograms of each chromosome; (b): TE density across the whole genome; (c): centromere regions; (d-h): normalized reads density (100 kb window) from 0, 3, 8, 12, 15, and 18 DPA, respectively; (i): gene-enriched regions.

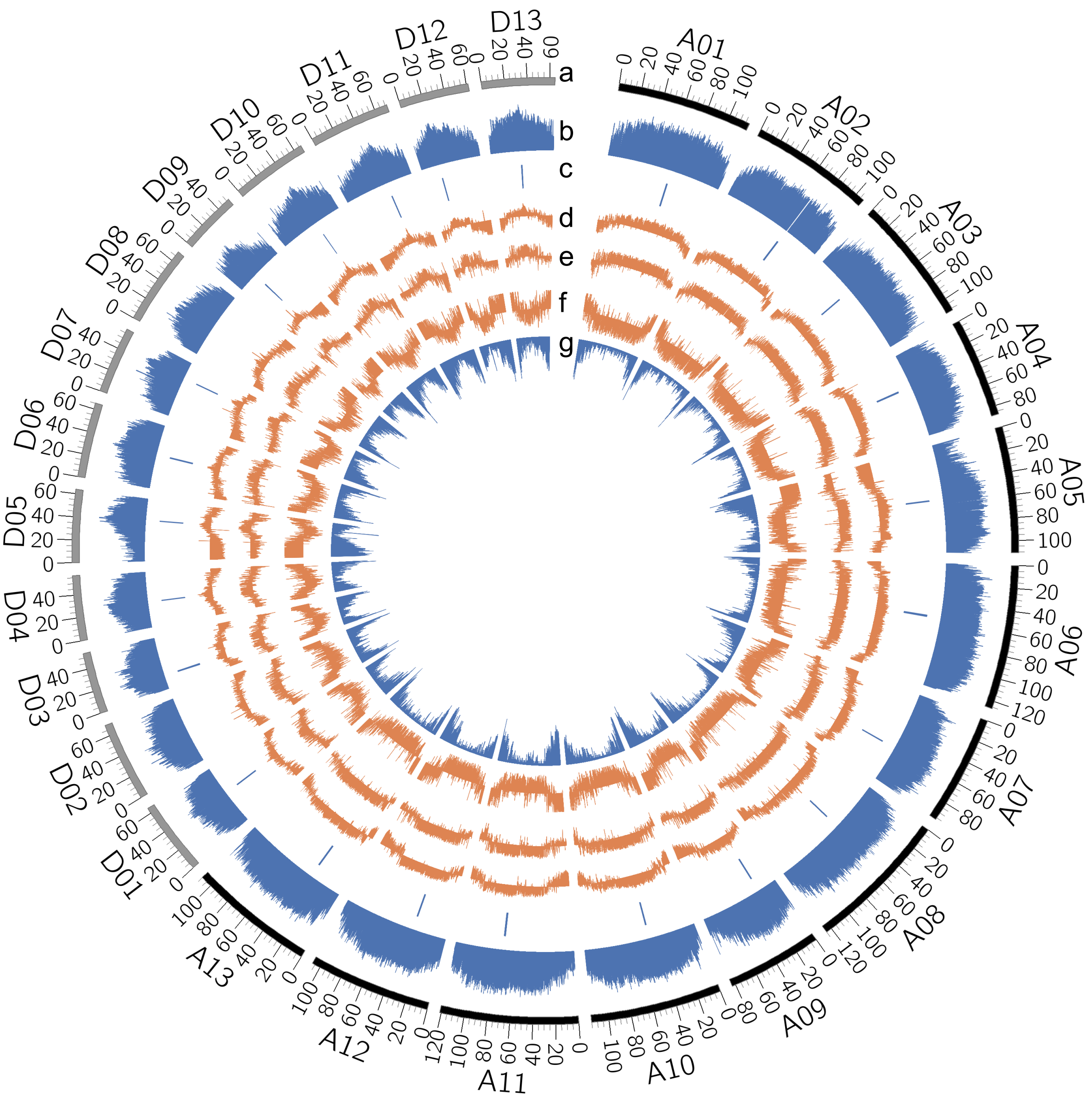

Figure S4. Distribution changes of DNase-seq, H3K4me3 and H3K27me3 along Chromosomes between 0 DPA and 8 DPA.

Genome-wide visualization the fold change of DNase-seq reads density(d), H3K4me3 reads density (e), H3K27me3 reads density (f) in cotton and aligned against other genomic features. (a): the ideograms of each chromosome; (b): TE density across the whole genome; (c): centromere regions; (g): gene-enriched regions.

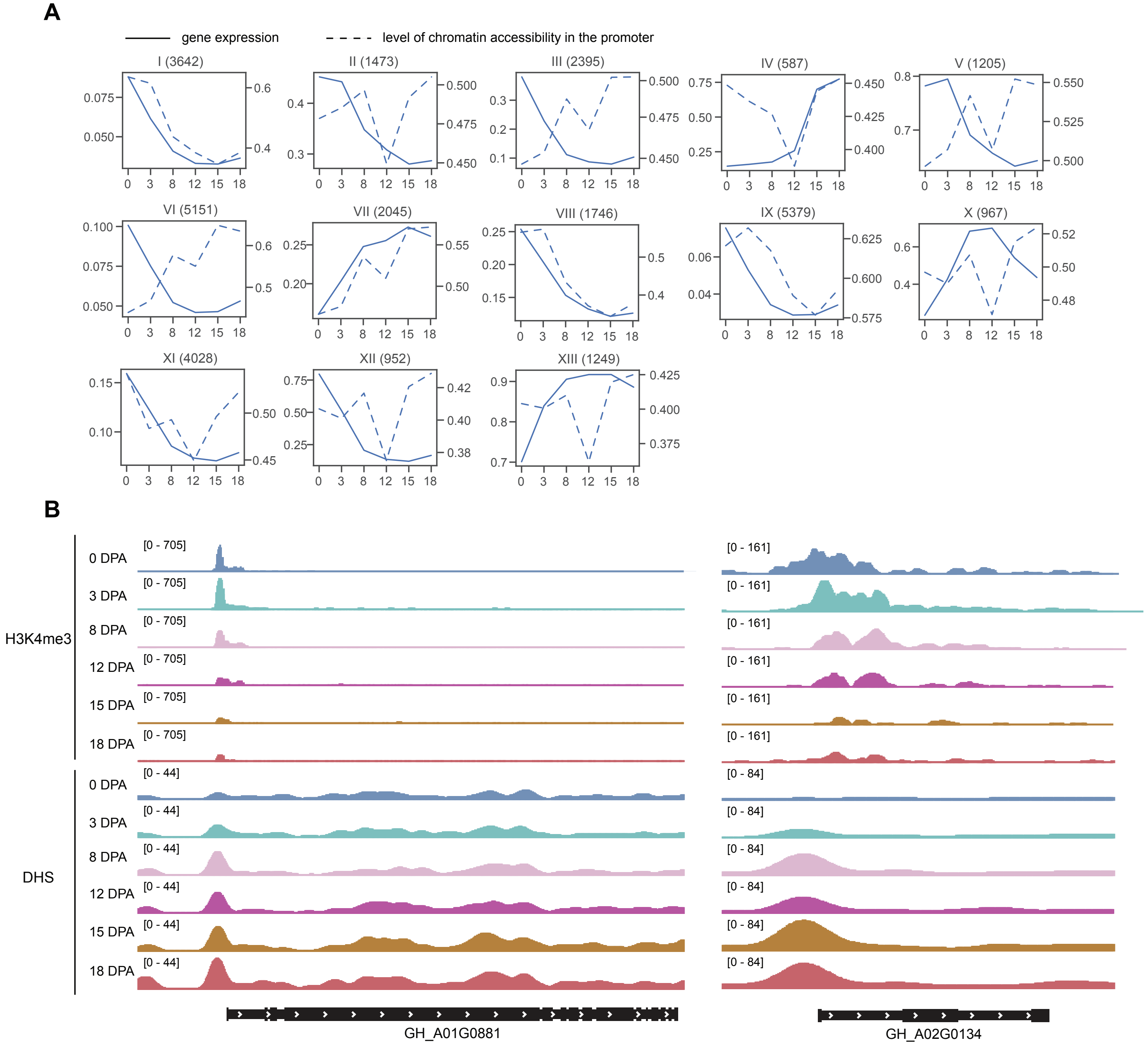

Figure S5. Relative transcript level and promoter DHSs changes for cluster I-VIII genes.
(A) Relative transcript level and promoter DHSs changes for cluster I-VIII genes.
(B) The H3K4me3 and DNase-seq tracks for the cluster III (left) and VI (right) gene are shown

A

		up	down
8 VS. 0	DEG	10390	7938
	DOCR	6471	15223
12 VS. 0	DEG	11757	9307
	DOCR	4262	9906
15 VS. 0	DEG	12317	9834
	DOCR	13159	23084
18 VS. 0	DEG	12253	9643
	DOCR	8377	14728
elongation VS. ovule	DEG	8903	5191
	DOCR	8906	17467

B

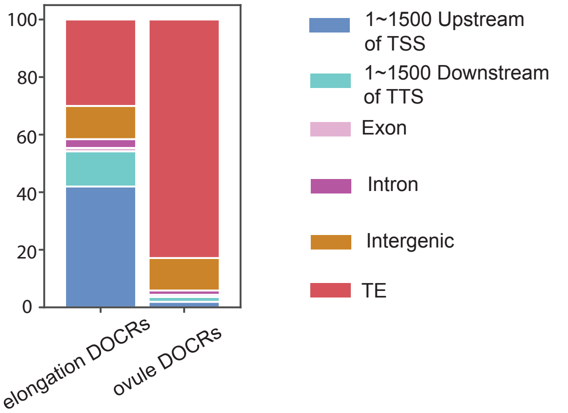

C

		all genes		TFs	
		peaks	genes	peaks	genes
Ovule	activating	2251	1552	222	143
	repressing	1375	986	77	50
Elongation	activating	2202	1714	234	173
	repressing	2804	2103	288	203

E

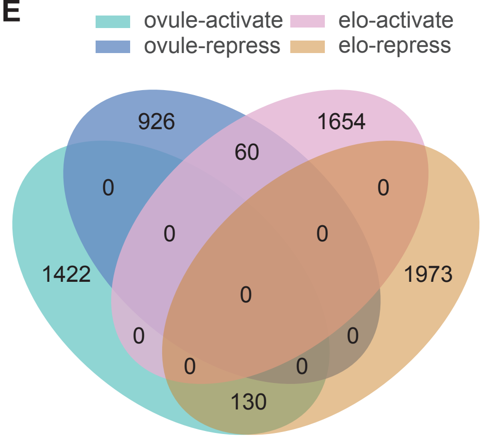

D

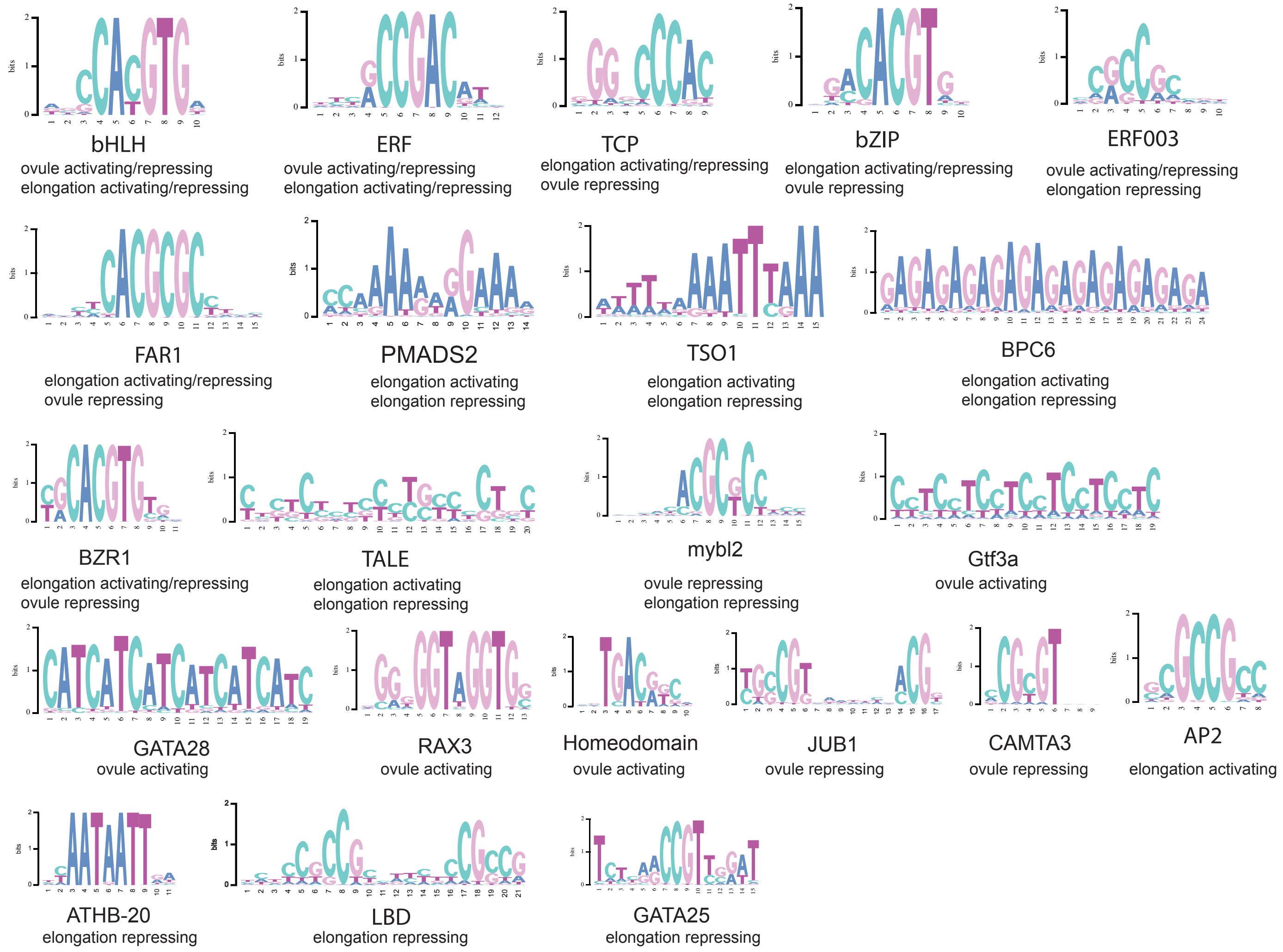

Figure S6. DOCRs configuration in the ovule and elongation.

(A) Summary of DEGs and DOCRs numbers between each elongation and ovule periods. up or down: upregulated or downregulated.

(B) The percentage of DOCRs falling in distinct regions of cotton genome.

(C) The table summarizes the number of activating or suppressing DOCRs associated with all DEGs or only differentially expressed TFs.

(D) representative TF motifs enriched in activating and repressing DOCRs in the ovule and elongation domains.

(E) Venn diagrams of activating and repressing DOCRs related genes during fiber ovule and elongation.

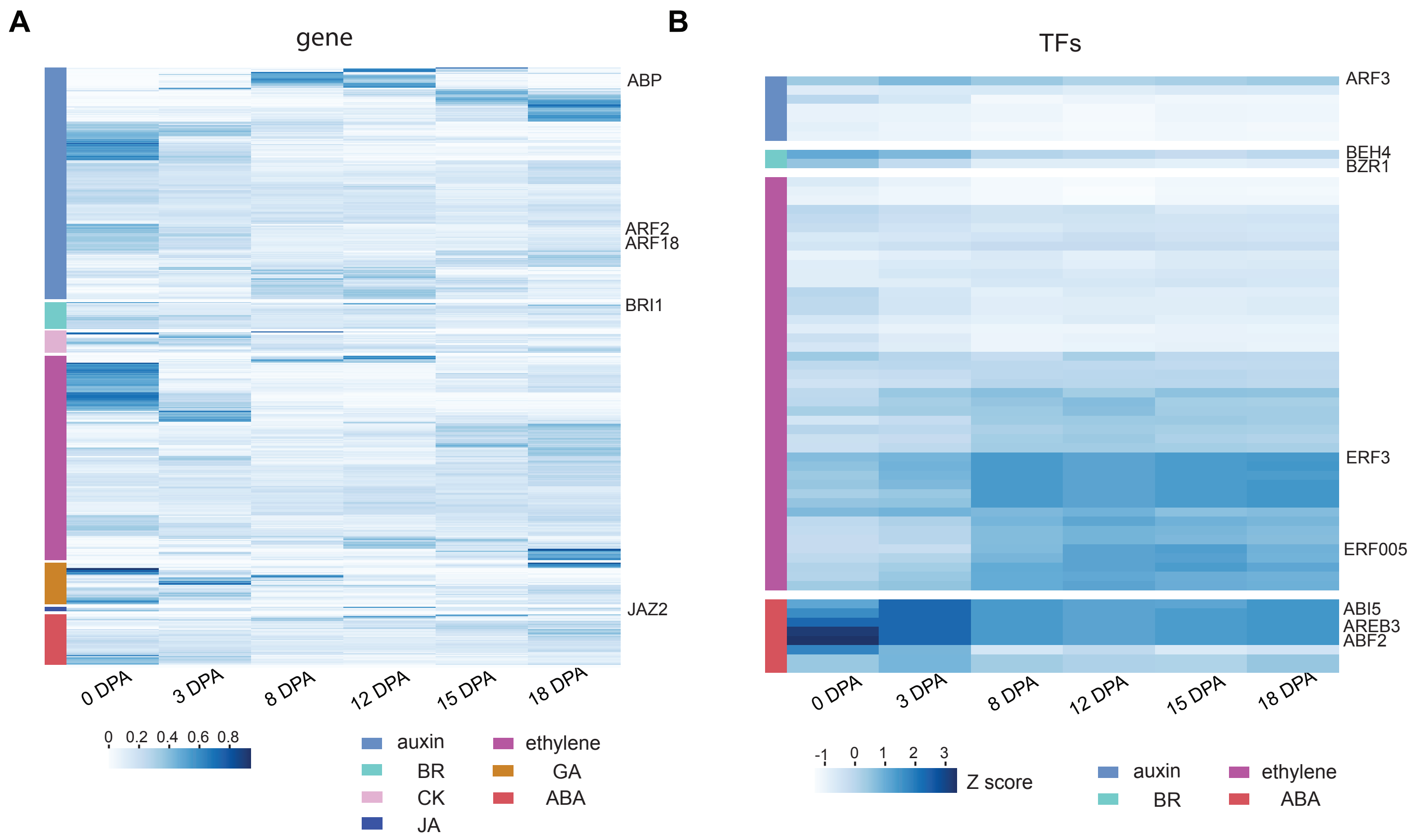

Figure S7. Dynamic of hormone activity during fiber development.

(A) The heatmap shows hormone related genes expression trends in the fiber development.

(B) The heatmap shows hormone related TFs activity score in the fiber development.

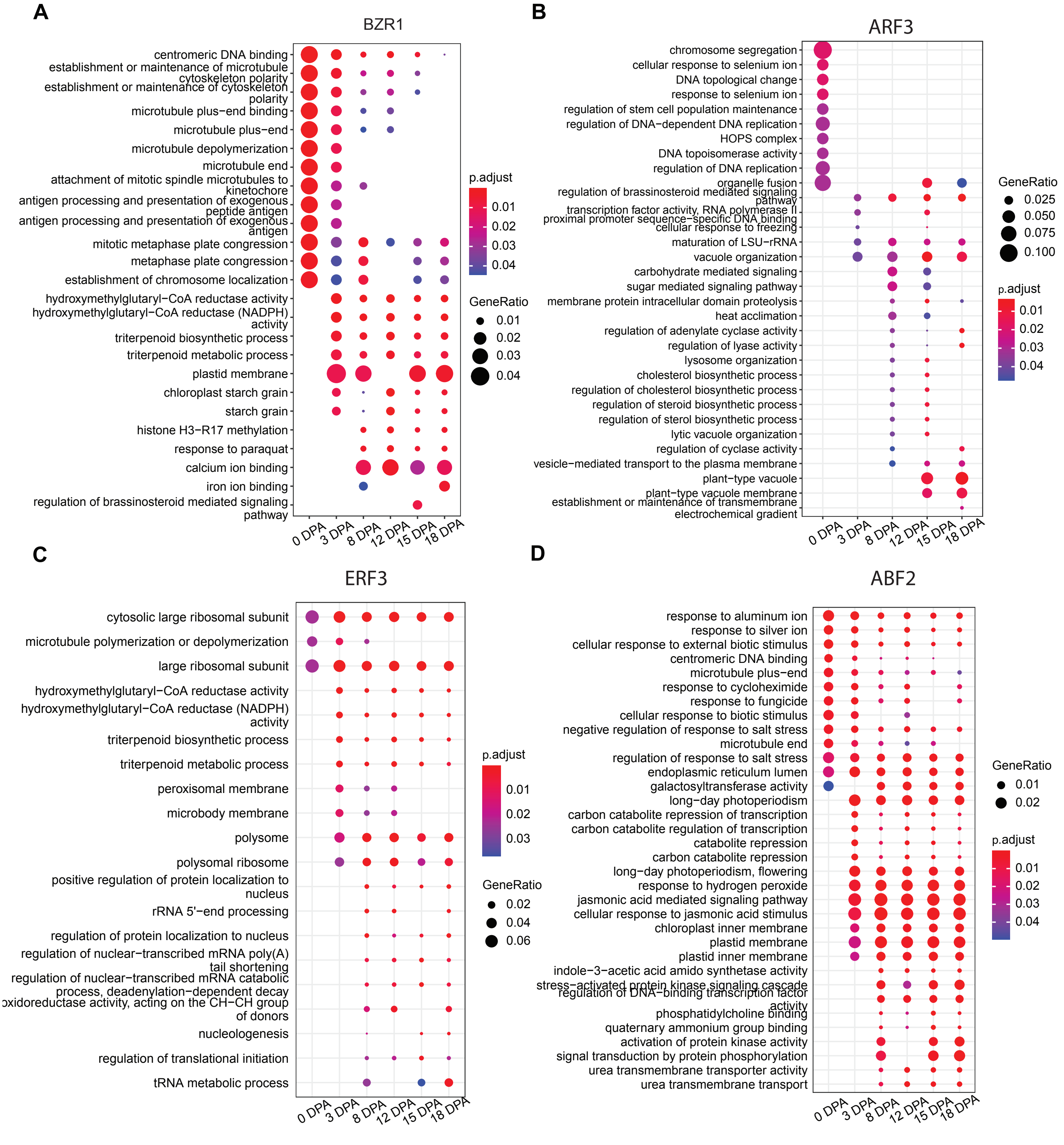

Figure S8. Gene Ontology (GO) terms with BZR1, ARF3, ERF3 and ABF2 downstream target genes, respectively.

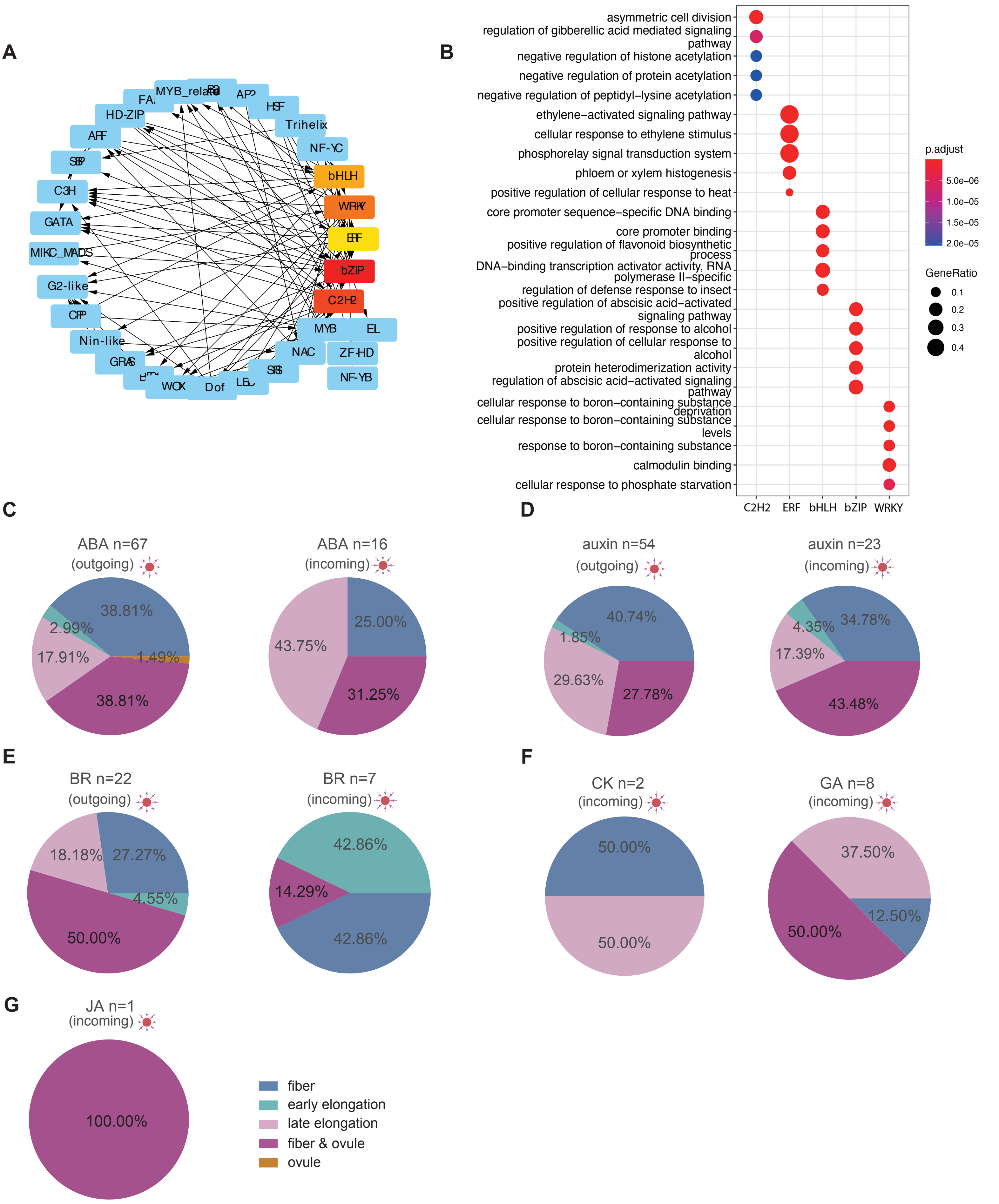
A
HD-ZIP
ARF
C3H
GATA
MIKC_MADS
G2-like
Nin-like
GRAS
WOX
Dof
Trihelix
NF-YC
bHLH
bZIP
C2H2
MYB
EL
NAC
ZF-HD
NF-YB
B
asymmetric cell division
regulation of gibberellic acid mediated signaling pathway
negative regulation of histone acetylation
negative regulation of protein acetylation
negative regulation of peptidyl−lysine acetylation
ethylene−activated signaling pathway
cellular response to ethylene stimulus
phosphorelay signal transduction system
phloem or xylem histogenesis
positive regulation of cellular response to heat
core promoter sequence−specific DNA binding
core promoter binding
positive regulation of flavonoid biosynthetic process
DNA−binding transcription activator activity, RNA polymerase II−specific
regulation of defense response to insect
positive regulation of abscisic acid−activated signaling pathway
positive regulation of response to alcohol
positive regulation of cellular response to alcohol
protein heterodimerization activity
regulation of abscisic acid−activated signaling pathway
cellular response to boron−containing substance deprivation
cellular response to boron−containing substance levels
response to boron−containing substance
calmodulin binding
cellular response to phosphate starvation
C2H2
ERF
bHLH
bZIP
WRKY
p.adjust
5.0e−06
1.0e−05
1.5e−05
2.0e−05
GeneRatio
0.1
0.2
0.3
0.4
C
ABA n=67
(outgoing)
38.81%
2.99%
17.91%
1.49%
38.81%
ABA n=16
(incoming)
25.00%
43.75%
31.25%
D
auxin n=54
(outgoing)
40.74%
1.85%
29.63%
27.78%
auxin n=23
(incoming)
4.35%
34.78%
17.39%
43.48%
E
BR n=22
(outgoing)
18.18%
27.27%
4.55%
50.00%
BR n=7
(incoming)
42.86%
14.29%
42.86%
F
CK n=2
(incoming)
50.00%
50.00%
GA n=8
(incoming)
37.50%
12.50%
50.00%
G
JA n=1
(incoming)
100.00%
fiber
early elongation
late elongation
fiber & ovule
ovule

Figure S9 Dynamics in transcription factor regulation in regulatory networks

(**A**) Identification of hub TF families in TF families network merged in all periods. Top 5 TF families (C2H2, ERF, bHLH, bZIP and WRKY) were identified by cytoHubba.

(**B**) Gene Ontology (GO) terms with genes of hub TF families

(**C-G**) Changes in regulation patterns during ovule and fiber development in outgoing and incoming connections

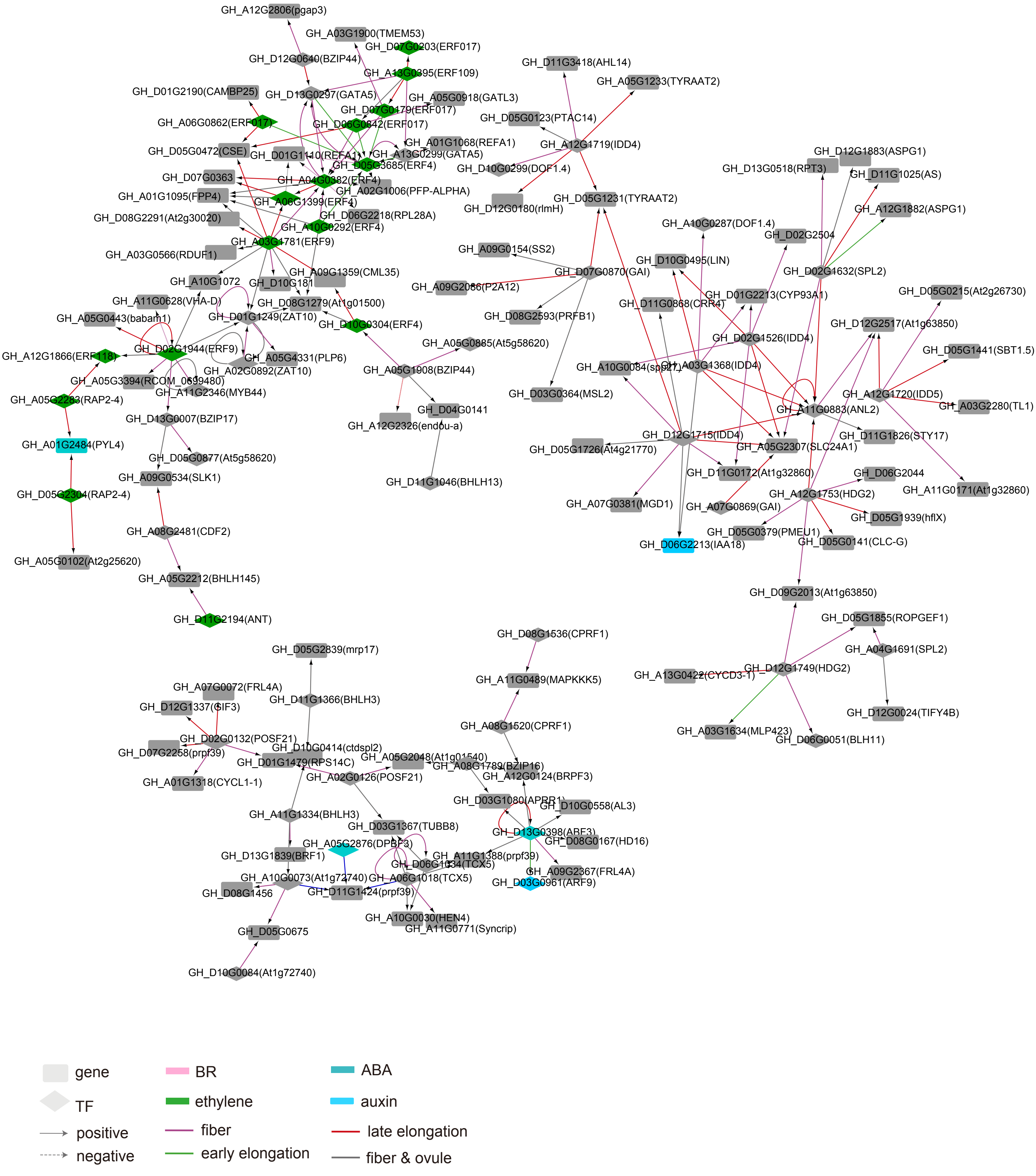

Figure S10 Subnetworks of hub TFs.

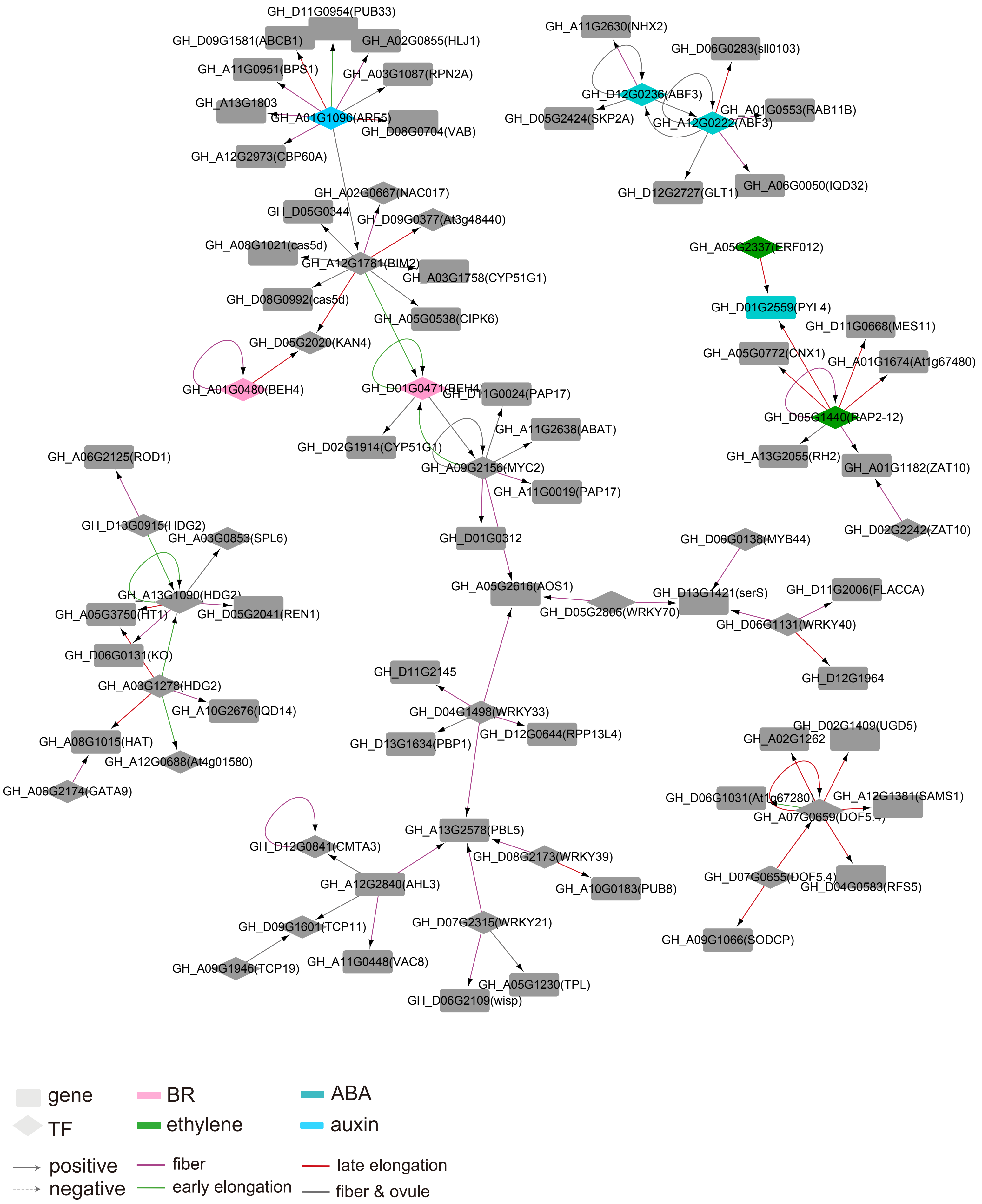

Figure S11 Subnetworks of hub TFs.

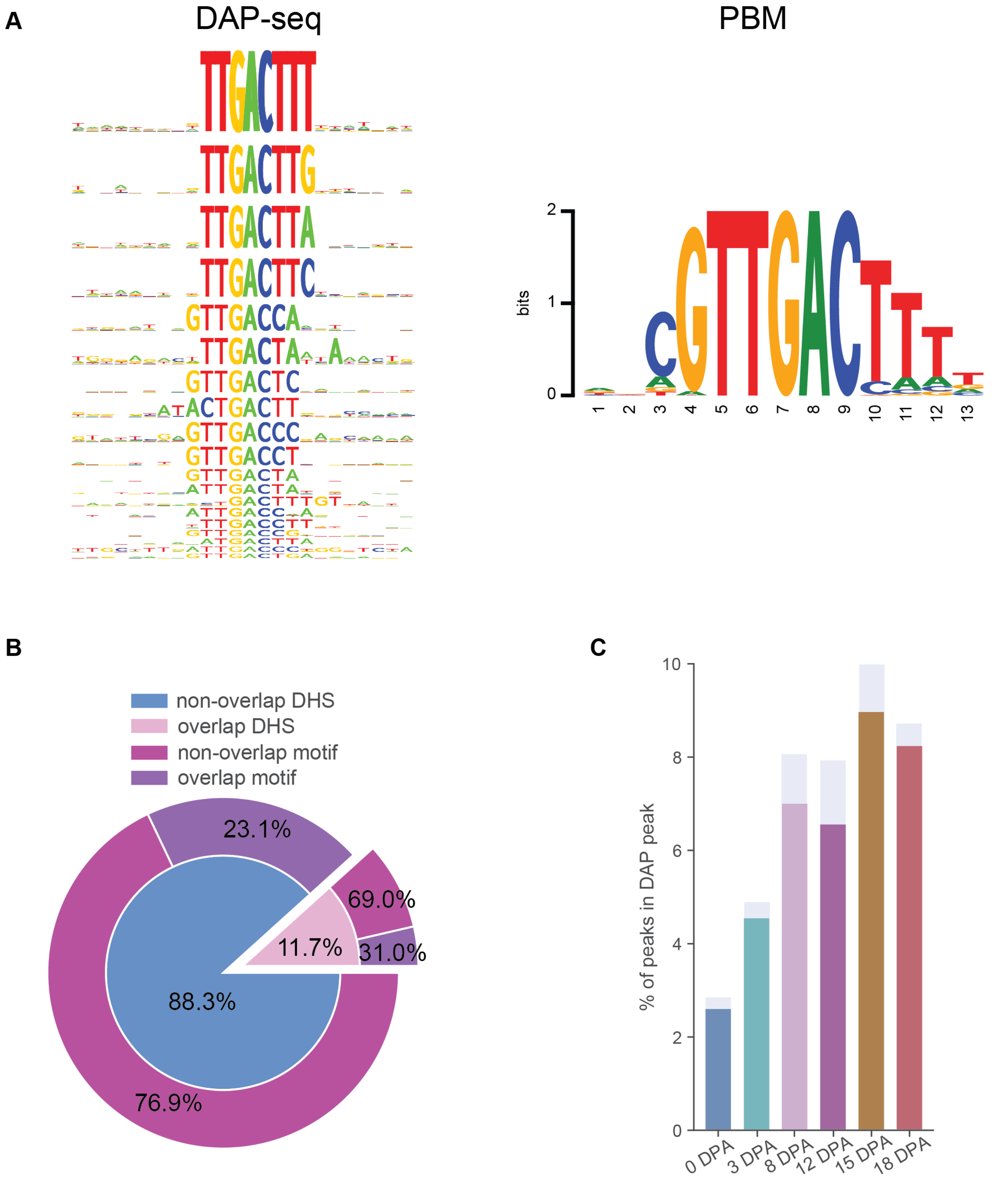

Figure S12. The relationship between DAP-seq and DHSs of fiber development stage.

(A) Motifs derived from DAP-seq match a published WRKY46 motif.

(B) The inner circle represents the overlapping ratio between DAP peak and DHSs, the outer circle represents the overlapping ratio between DAP peak and WRKY46 motif binding site.

(C) The overlapping ratio between DHSs and WRKY46 binding sites during fiber development. The y-axis represents the ratio of the overlapping number between DHSs and WRKY46 binding sites to DAP peak. The shaded portion represents the relative number of DHSs at each stage.

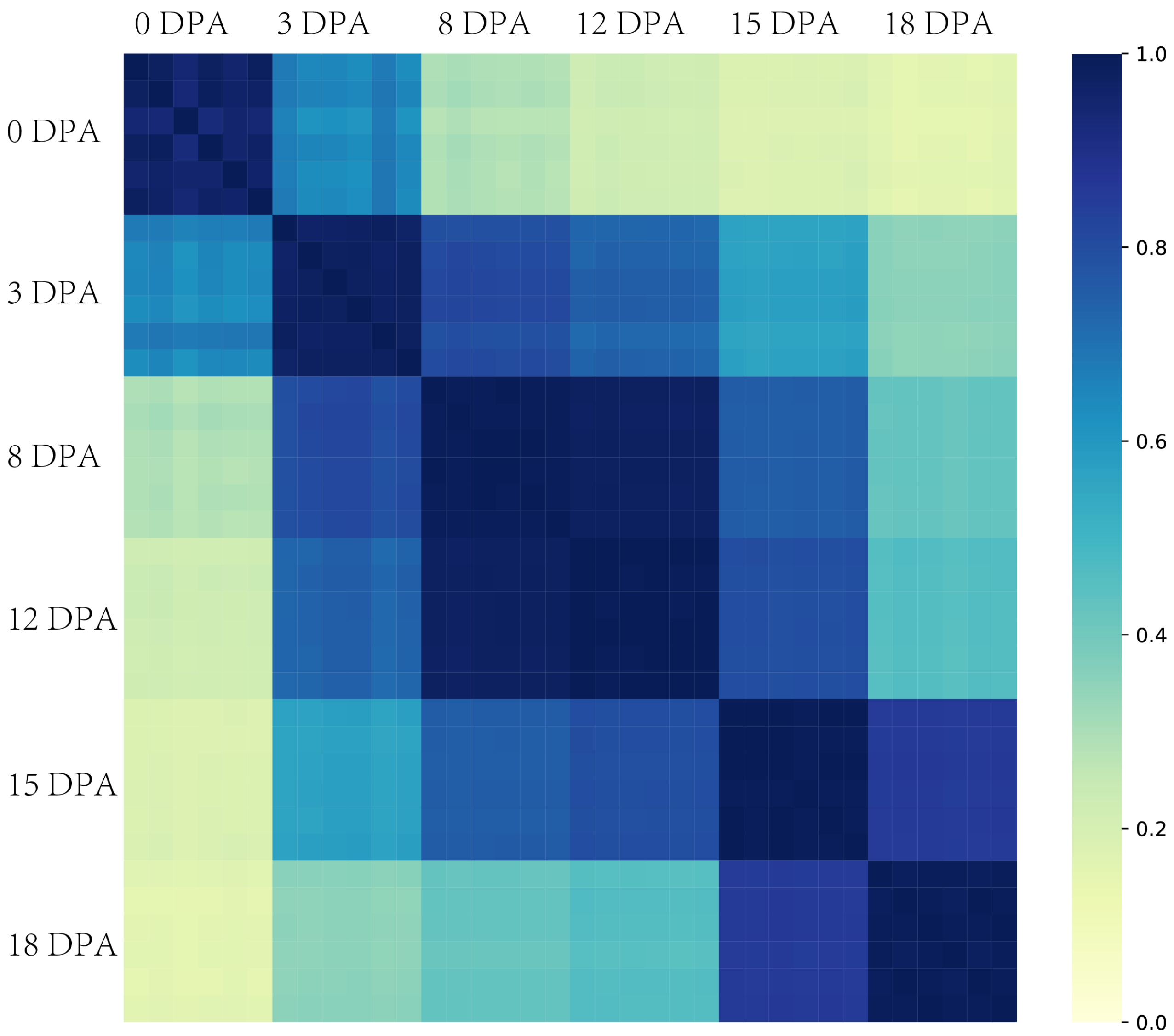

Figure S13. RNA-seq data correlation among different biological replicates and different technical replicates. The genes FPKM were used to calculate the Spearman Correlation Coefficient between replicates.
